# Cold climate adaptation is a plausible cause for evolution of multicellular sporulation in Dictyostelia

**DOI:** 10.1038/s41598-020-65709-3

**Published:** 2020-05-29

**Authors:** Hajara M. Lawal, Christina Schilde, Koryu Kin, Matthew W. Brown, John James, Alan R. Prescott, Pauline Schaap

**Affiliations:** 1 School of Life Sciences, University of Dundee, Dundee, DD1 5EH UK; 2 Dundee Imaging Facility, University of Dundee, Dundee, DD1 5EH UK; 3 Department of Biological Sciences, Mississippi State University, Mississippi State, MS USA

**Keywords:** Biological techniques, Ecology, Evolution

## Abstract

Unicellular protozoa that encyst individually upon starvation evolved at least eight times into organisms that instead form multicellular fruiting bodies with spores. The Dictyostelia are the largest and most complex group of such organisms. They can be subdivided into 4 major groups, with many species in groups 1–3 having additionally retained encystment. To understand fitness differences between spores and cysts, we measured long-term survival of spores and cysts under climate-mimicking conditions, investigated spore and cyst ultrastructure, and related fitness characteristics to species ecology. We found that spores and cysts survived 22 °C equally well, but that spores survived wet and dry frost better than cysts, with group 4 spores being most resilient. Spore walls consist of three layers and those of cysts of maximally two, while spores were also more compacted than cysts, with group 4 spores being the most compacted. Group 4 species were frequently isolated from arctic and alpine zones, which was rarely the case for group 1–3 species. We inferred a fossil-calibrated phylogeny of Dictyostelia, which showed that its two major branches diverged 0.52 billion years ago, following several global glaciations. Our results suggest that *Dictyostelium* multicellular sporulation was a likely adaptation to a cold climate.

## Introduction

The emergence of multicellular life forms was a major event in the history of life. The three most familiar transitions to multicellularity yielded the animals, plants and fungi. These organisms emerge from a spore or fertilized egg and achieve multicellularity by repeated cell divisions with the cells remaining together after each division. However, more common are forms that multiply as single cells while feeding, but aggregate to form spore-bearing multicellular structures when food runs out. This type of so-called sorocarpic multicellularity evolved independently in myxobacteria in prokaryotes, and at least seven times in different divisions of eukaryotes^1–4^. Dictyostelid social amoebas display the most sophisticated form of sorocarpic multicellularity with up to four somatic cell types^4^. *Dictyostelia* evolved from unicellular Amoebozoa, which, like many protists, survive starvation by encapsulating individually to form a dormant cyst.

**Figure 1:**
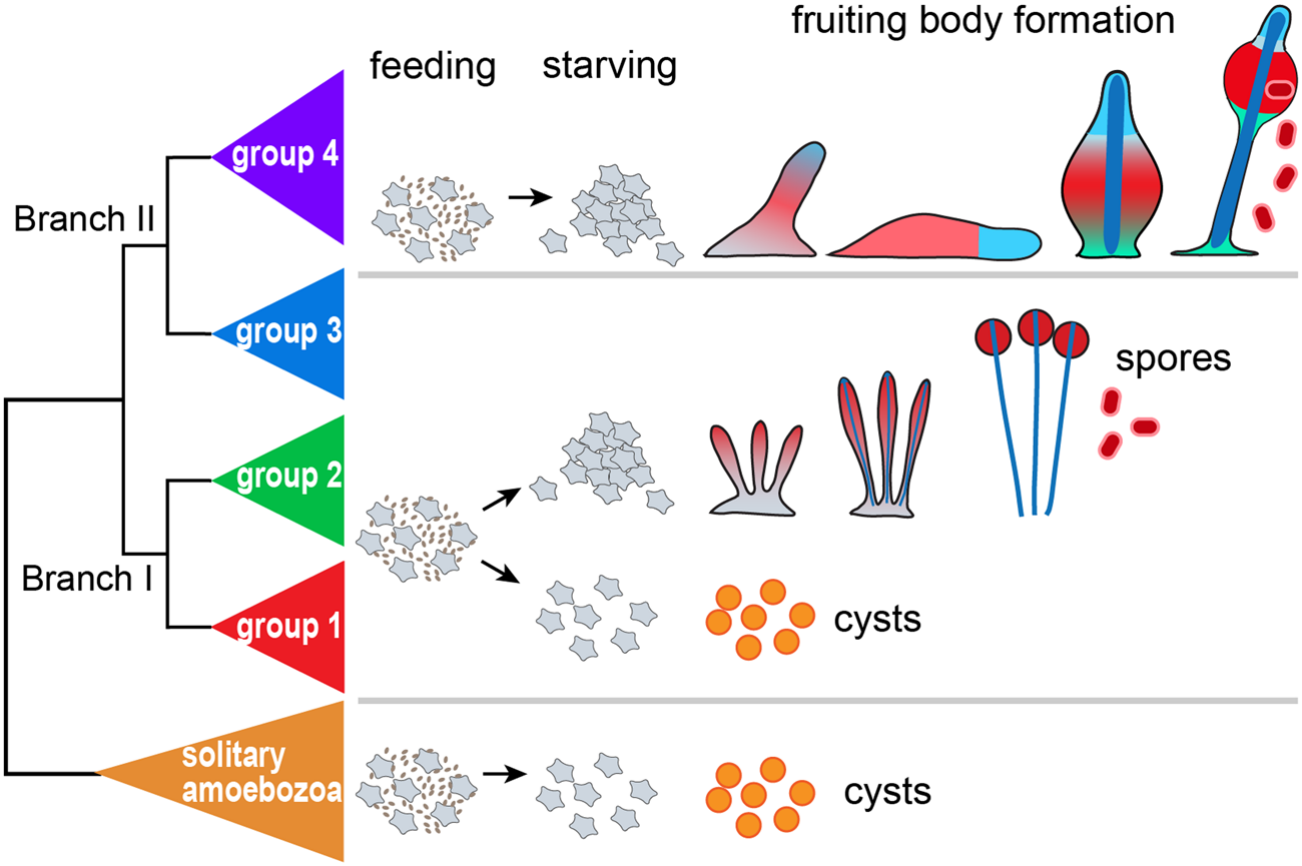
Encystation and sporulation in Amoebozoa. Solitary Amoebozoa usually feed on bacteria and differentiate individually into walled dormant cysts when food runs out. Many Dictyostelia in groups 1, 2 and 3 retained this strategy, but, when starved, preferentially aggregate to construct multicellular fruiting bodies with walled stalk cells (blue) and walled dormant spores (red). Group 4 Dictyostelia have lost encystation, but gained more cell types to support the stalk and spore head.

The ~150 known species of Dictyostelia can be subdivided into two branches, each containing two major and some minor groups^5–7^. Phylogenetic comparative analysis showed that the last common ancestor (LCA) of Dictyostelia erected small fruiting structures directly from aggregates, while retaining encystation as an alternative survival strategy. It formed a cellular stalk by transdifferentiation of prespore cells at the fruiting body tip (Fig. 1). This phenotype persisted up to the LCAs of groups 1, 2 and 3. The LCA to group 4 in branch II lost encystation and formed migrating slugs, inside which cells pre-differentiated in correctly proportioned stalk and spore precursors. Group 4 also evolved novel somatic cells to support the stalk and spore mass^8,9^. This ability to assign a proportion of cells to specialized roles that support rather than propagate the organism is the most important asset of multicellularity. However, because it comes at a cost to the number of propagating cells, there must be a distinct advantage to sporulating in fruiting bodies as opposed to encysting individually. Both spores and cysts have cellulosic walls, but differ in shape. Cysts are always globose, while spores are with a few exceptions elliptical or capsule-shaped, likely to facilitate closer packing in the spore head.

Sporulation in multicellular fruiting structures is commonly thought to aid wide-spread dispersal of organisms, as is the case for fungi with their hydrophobic spores. However, this is less clear for the Dictyostelia, whose hydrophilic spores are not carried by wind. Dictyostelia have been isolated from soils in all climate regions, except deserts^10^ and were previously estimated to have evolved from 600 million to 1 billion years ago^5,11^. The animal kingdom was then still ocean-bound and small soil invertebrates could therefore not have contributed to spore dispersal. Other agents like rain and snowmelt also disperse the soil-bound cysts of unicellular protozoa, making improved dispersal an unlikely trigger for multicellular sporulation in Dictyostelia.

To evaluate a possible fitness advantage of spores over cysts and among spores of Dictyostelids from different taxon groups, we subjected spores and cysts to storage regimes at different temperatures to mimic local climates, and measured survival rates for up to a year. To gain insight into structural differences between spores and cysts that could be related to fitness, we investigated spore and cyst ultrastructure by electron microscopy. Our results show that spores are particularly well adapted for survival just above and below 0 °C. This feature is most prominent in group 4, and correlates with group 4 spores combining a relatively thick wall with signs of extreme dehydration. Survey of global distribution data of species revealed that group 4 species were more frequently isolated from arctic and alpine regions than those in other groups. To assess whether climate change might have necessitated the evolution of a frost-resistant dormant stage, we inferred the divergence time of Dictyostelia from a novel dataset. Our combined results suggest that Dictyostelid multicellular sporulation may have been triggered by global cooling during the neoproterozoic “snowball earth” episodes.

## Results

### Spores survive frost better than cysts

**Figure 2:**
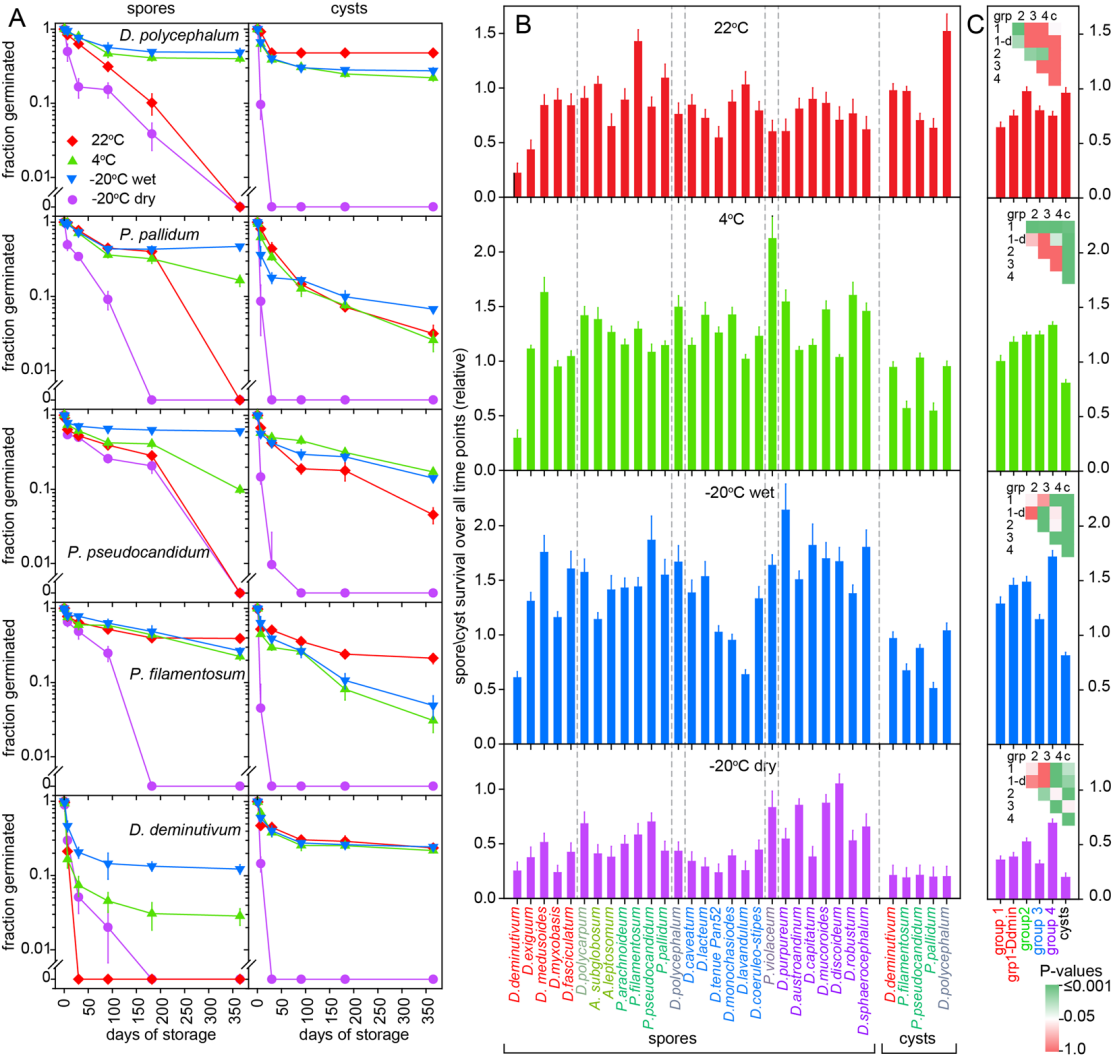
Spore and cyst survival. (**A**) *Spore and cyst survival*. Spores and cysts were stored for up to a year at 22 °C, 4 °C, and −20 °C wet or dry. At the indicated time points, spores and cysts were clonally plated with food bacteria and emerging plaques were counted. Means and SD of triplicate measurements. Survival curves for another 23 species are shown in Fig. S1. (**B**) *Relative spore and cyst fitness*. For all survival curves, the fraction of surviving spores or cysts at each time point was normalized to the averaged spore survival at that time point for all species and conditions. The normalized values were then averaged per species and condition over the six time points (See Data1_Sporefitness.xlsx) and are shown here with standard errors. (**C**) T*axon group specific differences*. Relative spore and cyst fitness values for each treatment were pooled per taxon group (grp) for spores or over all cysts (c). Means and SE were calculated and significant differences between pools were assessed by ANOVA on ranks. A matrix of P-values for pairwise comparisons is shown (see Data1_Sporefitness.xlsx, sheet 7).

To assess differences in spore and cyst fitness, spores and cysts were stored wet for a year at 22 °C, 4 °C and −20 °C to reflect summer and winter temperatures, and at −20 °C after desiccation to mimic dry frost at high altitudes. Spore survival was tested on five species from each of the four major taxon groups and one species each for the minor groups, and cyst survival was tested on five species that readily form cysts. The survival curves for five species that form both spores and cysts show that cysts survived 20 °C similar or better than spores of the same species, but that cysts lost viability entirely within a few days of dry frost, which at least a proportion of spores withstood for 3 or more months. Apart from *D. deminutivum*, a species with very small spores, spores also survived 4 °C and wet frost better than cysts (Fig. 2A). Across the phylogeny, spores generally survived better at 4 °C and wet frost than at 22 °C or dry frost (Fig. 2B). While there is considerable variation in spore survival rates between species in the same taxon group, those in groups 1 and 3 show on average the lowest, and those in group 4 the highest survival at wet and dry frost (Figs. 2B,C, S1 and S2).

### Relationships between spore and cyst survival and ultrastructure

**Figure 3:**
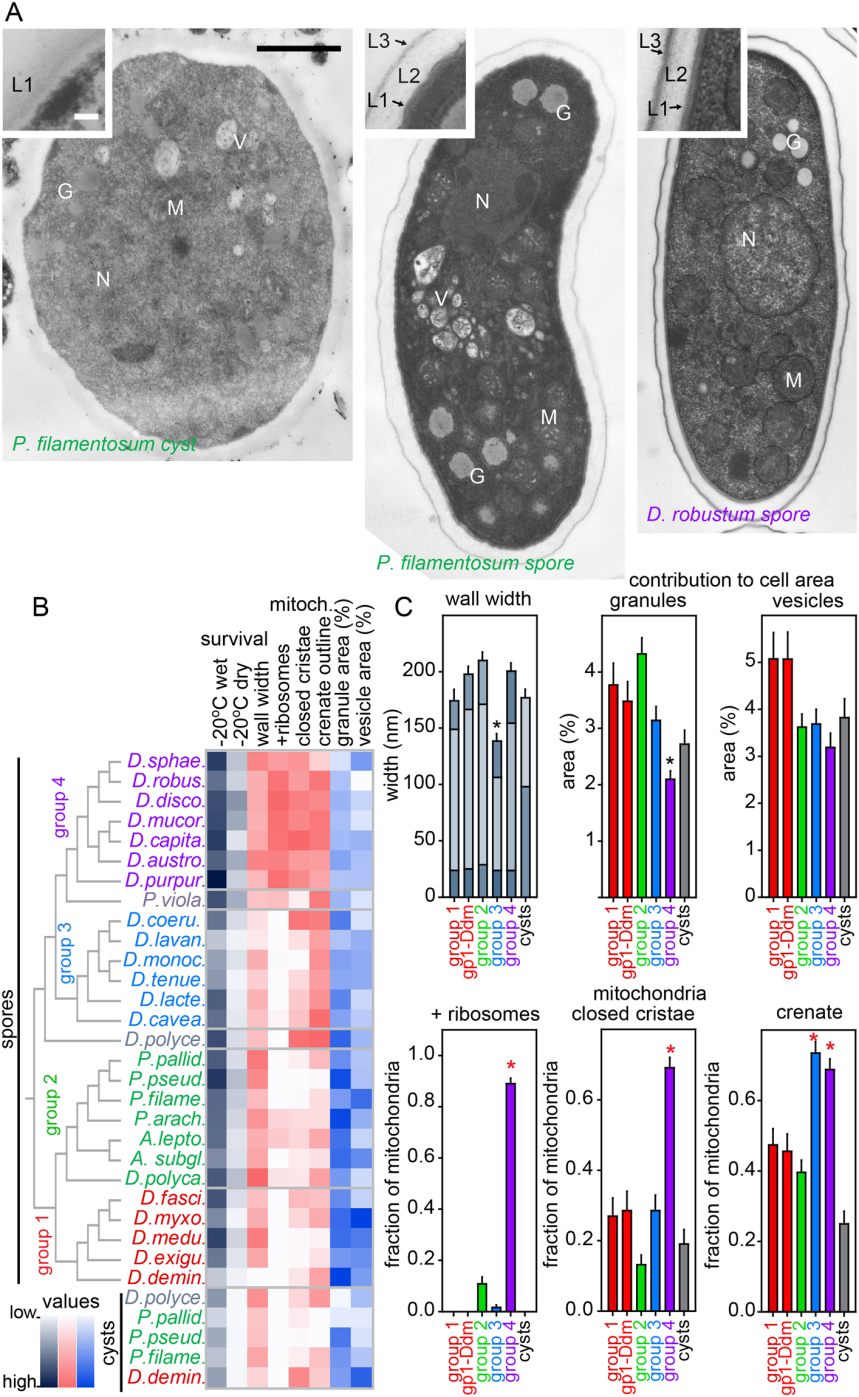
Relationships between spore/cyst fitness and ultrastructure. (**A**) *Spore and cyst ultrastructure*. TEM images were prepared of cysts and spores of 34 species (see Figs. S3–S7). An example cyst and spore of the group 2 species *P. filamentosum* and a spore of the group 4 species *D. robustum* are shown here (Bar: 1 µm, N: nucleus, M: mitochondria, V: vesicles, G: granules). The inset shows a section of the cell wall with its discernable layers (L), bar: 0.1 µm. (**B**) *Correlated features*. 20 ultrastructural features were quantitated across 10 spores or cysts for all species (see Figs. S8–S12). Correlations were determined between spore/cyst survival and ultrastructural features (Data4_Correlations.xlsx, Table S3). Values of features that are positively (red) or negatively (blue) correlated with spore/cyst survival at −20 °C (grey) are shown as heat maps. Phylogenetic tree as previously inferred^7^. (**C**) *Group specificity*. Ultrastructural features that are correlated with spore fitness are averaged per taxon group. Values annotated with red or black asterisks are significantly higher or lower than the others, respectively (P < 0.05). For walls, the widths of individual wall layers are shown as stacked bars.

To understand why spores survive long term storage better than cysts and why some spores are more frost resistant than others, we imaged spores and cysts of 34 species by transmission electron microscopy (TEM) (Figs. 3A, S3–S7). Spores are highly condensed with the organelles packed closely together. The spore wall typically consists of two narrow dense layers that flank a broader more transparent layer. The cyst wall is less structured with only one or two layers and the cyst cytoplasm is less condensed. We quantitated 20 ultrastructural features, such as wall structure and width, and the morphology, distribution, size, and relative combined cross-section area of organelles (Figs. 3B, S8–S12). Many features were not significantly correlated with spore or cyst fitness (Table S3), although they may be of taxonomic value. However, frost survival was positively correlated with wall thickness and with mitochondria showing closed cristae, a crenate outline and a fringe of ribosomes. Frost survival was negatively correlated with a relatively large contribution of granules and vesicles to the cytosol cross-section area (Fig. 3B, Table S3). Low fractional contribution of vesicles and granules as well as ribosome-fringed crenate mitochondria with closed cristae are typically found in group 4 species (Fig. 3A,C,D *robustum*) and likely represents high compaction of the spore content by loss of water. Group 3 also has crenate mitochondria, but has the thinnest spore walls, whereas group 2 has the thickest walls, but none of the other features. We therefore hypothesize that group 4 spores owe their frost resistance to a combination of low water content and thick walls. Apart from ultrastructure, many other factors, such as the molecular composition of the spore or cyst wall or the presence of stored nutrients and cryoprotectants are also likely to affect spore and cyst fitness, but even when known, such factors require extensive experimentation to quantitate across species.

### Relationships between climate and spore- and cyst fitness

**Figure 4:**
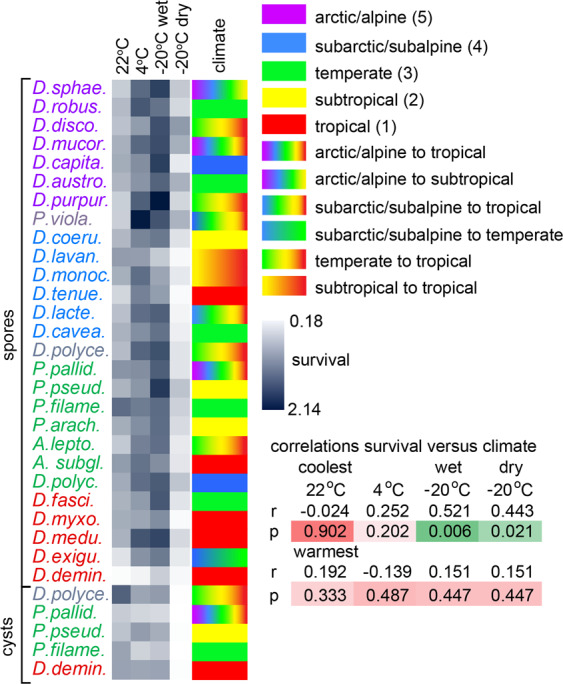
Relationship between spore fitness and climate at site(s) of origin. Climate or climate ranges at sites from which Dictyostelia were isolated were retrieved from literature data (see Data4_Ecology.xlxs) and ranked 1–5 from tropics to arctic/alpine. Rankings for either the coolest of warmest climate of origin were correlated with spore survival under different storage conditions as shown in Fig. 2B, using Spearman rank order. Correlation coefficients (r) and P-values (p) are shown.

Dictyostelia are most abundant in the wet (sub)tropics and decline in number and species diversity at increasing latitude and altitude^10,12^. To assess whether the differences in cold resistance between species are related to their natural habitat, we compiled literature data on climate at sites of species isolation (Data4_Ecology.xlsx) and compared these data with spore and cyst survival. Figure 4 shows that group 4 species with their more cold-resistant spores tend to be more frequently isolated from arctic/alpine to temperate zones than species from the other taxon groups. By comparing a simple ranking of coldest climate of isolation from 5 for arctic/alpine to 1 for tropics with survival at the different tested conditions, no significant correlations were found between survival at 22 °C or 4 °C and the climate of origin. However, survival in wet or dry frost was positively correlated with the coolest species habitats. There was no correlation between spore survival and the warmest species habitats (Fig. 4).

A broader survey of ecological data from most known Dictyostelia confirms the group 4 resilience to cold climates with 19% of species being isolated from alpine to arctic regions, as opposed to 0% for groups 1 and 3, and 4% for group 2 (Fig. S13, Table 1). Group 4 species are also more frequently isolated from the subarctic and temperate zones than species from the other groups, which prefer the (sub)tropical zones. Evidently, the increased frost-resistance of group 4 spores allowed them to colonize colder habitats. Equally, the large difference in frost resistance between cyst and spores may indicate ecological stressors that caused unicellular Amoebozoa to convert to multicellular sporulation.

**Table 1:** Coldest climates from which species have been isolated.

	% of species from each group				
	arctic/alpine	subarctic/ subalpine	temperate	subtropical	tropical
group 4	19	15	52	4	11
group 3	0	8	31	46	15
group 2	4	4	33	22	37
group 1	0	18	35	0	47

For each taxon group, the percentage of species isolated at the indicated five climate zones was determined from data presented in Data4_Ecology.xlsx and Fig. S13. For species isolated from a range of climate zones, the coldest zone was used.

### Paleontological climate at the emergence of multicellular sporulation

**Figure 5:**
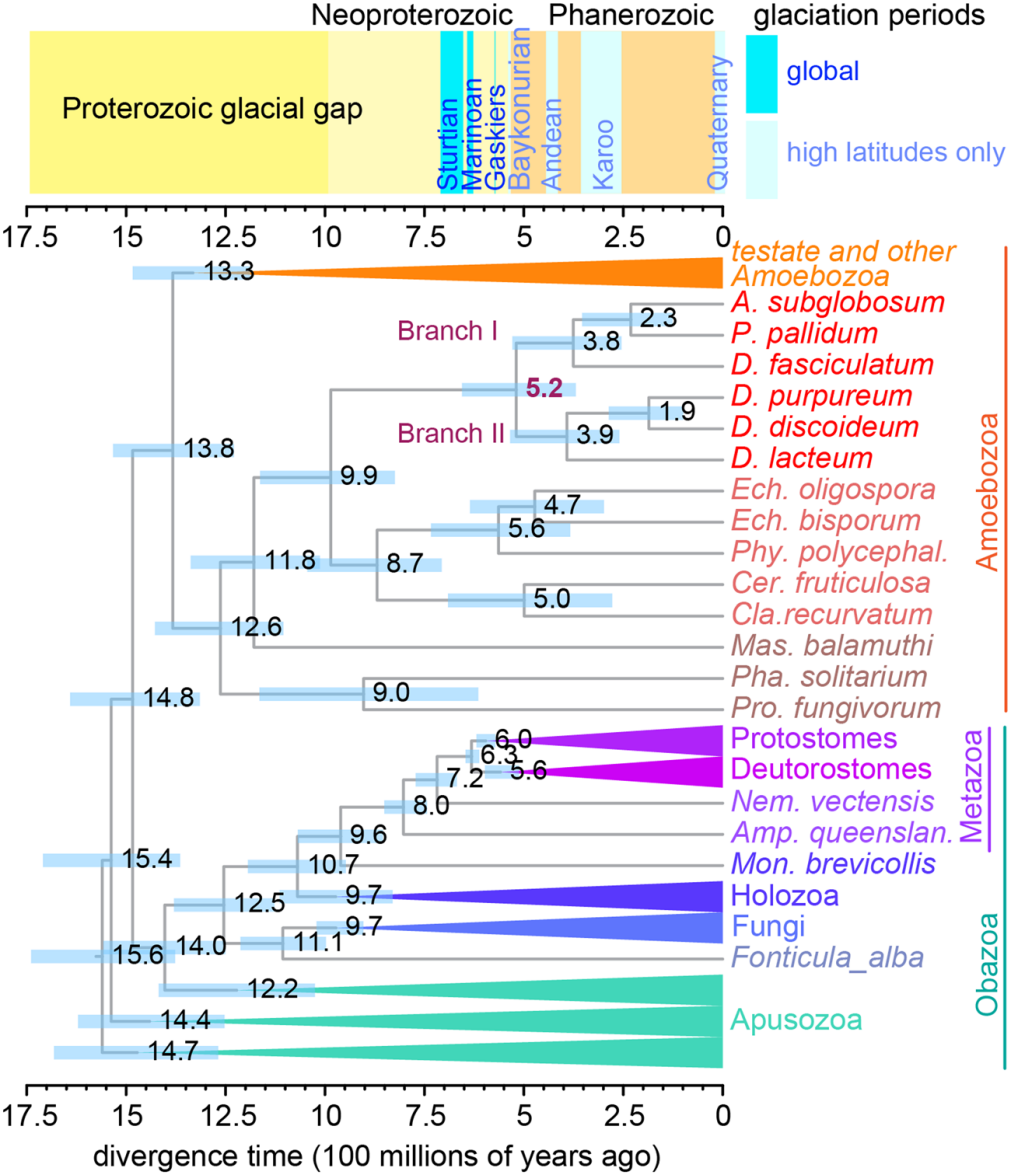
Emergence of dictyostelid multicellularity. Divergence time estimates were inferred using MCMCtree (see Methods) from a phylogeny of 240 concatenated protein sequences over 85 species of Amorphea, calibrated with a set of age calibration points of metazoan, fungal and testate amoebozoan fossils (Table S4). The posterior mean of the divergence times in hundred millions of years ago are shown at the tree nodes, with 95% Highest Posterior Density (HPD) intervals outlined by blue bars. Larger clades have been collapsed, but the full tree is shown in Fig. S14. The time scale of the proterozoic and phanerozoic glaciations is shown at the top.

An earlier phylogeny of 30 concatenated proteins, calibrated on an estimated divergence between the human and hydra lineages at 750 million years ago (mya), dates the split between the two major branches of Dictyostelia at 600 mya^11^. Many more genomes in both Opisthokonta and Amoebozoa, which together make up the Amorphea^13^ have since then become available, as well as fossil dating of testate arcellinid amoebas within Amoebozoa^14,15^. To obtain a more accurate estimate of the divergence time of the two major branches of Dictyostelia, we inferred a novel phylogeny using 240 concatenated proteins across 85 species of Amorphea, and calibrated this phylogeny on fossil dating of divergence between major branches of testate amoebas, Fungi and Metazoa under relaxed molecular clock models (Fig. S14, Fig. 5). The new time-tree estimates the divergence of branch I and II of Dictyostelia at 519 ± 140 mya. This date is roughly contemporary with the Cambrian radiation of animal phyla^16^ and follows the cryogenic period from 720–635 mya, during which two periods of complete global glaciation, known as “snowball earth” occurred^17^. The short Gaskiers glaciation at 580 mya likely did not achieve complete global coverage^18^, while the later glaciations in the Phanerozoic were restricted to higher latitudes^19^. The earliest diverging extant Dictyostelia already show highly organized stalk formation^9^, which suggests that “proto-Dictyostelia” evolved sporulating fruiting bodies with less complex stalks considerably earlier, but that these intermediate forms are either extinct or undiscovered. Earlier emergence of proto-Dictyostelia is supported by the very long branch (equating 470 my) that separates Dictyostelia from their closest unicellular relatives and brings the origin of multicellular sporulation closer to the onset of the cryogenic period.

## Discussion

To gain insight into plausible ultimate causes for *Dictyostelium* multicellularity, we searched for physiological differences that distinguish spores, which differentiate in fruiting bodies, from cysts, which differentiate directly from starving amoebas. Spores and cysts showed similar long-time survival at 22 °C, but cysts survived temperatures close to and below 0 °C worse than spores and survived dry frost very poorly. Across *Dictyostelium* taxon groups, group 4 spores performed best at surviving wet and dry frost, followed by spores in group 2. These results indicate that cysts are more suited for surviving starvation in summer or in (sub)tropical climates, while spores are adapted for surviving winters in higher latitudes and altitudes.

At the ultrastructural level, cysts are most different from spores in the structure of their cell wall, which consists at most of two poorly defined layers, in contrast to the three well-defined layers of the spore wall. The spore cytoplasm is also more condensed and this is particularly the case for group 4 spores, which show typical crenate mitochondria without visible cristae, as if they had collapsed into themselves. These mitochondria are also surrounded by a ring of ribosomes. Crenate mitochondria without ribosomes are also found in group 3 spores, but compared to the other groups, group 3 spores have rather thin walls. A combination of thick walls and compaction of the cytoplasm may therefore be factors that provide group 4 spores with their higher frost resistance.

The presence of distinctive polar granules was used earlier to distinguish species that use cAMP as attractant and do not form cysts from those that do form cysts and use other attractants^20^. The former are now known to belong to group 4^8^. Our ultrastructural study showed that polar granules are indeed quite prominent in group 1, 2 and 3 spores, but they are also present in cysts and in spores of group 4 (Figs. S3–S7). However, in group 4 spores, the granules are more dispersed throughout the cytoplasm and both their diameter and total contribution to the spore cross section area is less than in groups 1 to 3 (Fig. S9). This is the likely reason that they are indistinctive when viewed with light microscopy. In fact their reduced diameter and reduced total cross-section area is another hallmark of the compaction of the group 4 spore cytoplasm.

Comparison of long term frost resistance of spores with geographical distribution of the 29 investigated species showed a positive correlation between frost resistance of spores and species origins from higher latitudes and altitudes (Fig. 4). A broader analysis of 85 species with recorded climate of origin, revealed that only 15% of group 4 species was isolated from the (sub)tropics, while this was 47, 59 and 61% for group 1, 2 and 3 species, respectively. Five out of 27 group 4 species were recovered from alpine or arctic tundra against only 1 (*P. pallidum*) from the other 58 species. While investigating the distribution of Dictyostelia in the Himalayas, Hagiwara noted that only species without polar granules such as *D. brefeldianum, D. aureocephalum* and *D. mucoroides* (now known to reside in group 4) could be isolated from the alpine zone above 4000 m, while species with polar granules (groups 1–3) could also be recovered from the subalpine zones^21,22^. These combined results indicate that the improved frost resistance of group 4 spores allowed them to colonize extremely cold habitats, while overall the larger frost resistance of spores over cysts suggest that multicellular fruiting body formation may have been driven by adaptation to colder habitats or climate change.

To associate paleontological climate with the emergence of sporulation in Dictyostelia, we refined estimates of the time of split of the two major branches of Dictyostelia by inferring a fossil calibrated phylogeny of Amoebozoa and its sister group the Obazoa. The split was estimated to occur at 519 ± 140 mya, following periods of extreme glaciation from 720-580 mya. While not conclusive, the increased frost resistance of multicellular spores versus unicellular cysts and the correlation between spore frost-resistance and frost-prone species habitats combined with the paleontological record argues in favor of *Dictyostelium* multicellular sporulation having arisen as a response to global cooling.

While we propose that cold adapation could have triggered the evolution of multicellular sporulation, an obvious alternative would have been for cysts to become more frost-resistant. One possibility is that the stalk cells serve a second function in addition to lifting the spore mass. In *D. discoideum* at least 20% of aggregating amoebas are incorporated into the stalk, obviously at a cost to the number of propagating spores. The stalk cells undergo extreme autophagy while passing through the centre of the mass of maturing spores (Fig. 1). While this is thought to yield materials for construction of the stalk wall, it is well possible that these materials also diffuse to the maturing spores to become incorporated in the spore wall. The cysts would lack this additional source of wall materials.

The aggregation process that precedes fruiting body formation may well have been triggered by other environmental factors than climate. Escaping predation is a likely cause; when Dictyostelia become deprived of bacteria, it is likely that other protozoa in their habitat will be as well and then look upon the relatively small *Dictyostelium* amoebas as food. Aggregation of solitary starving amoebas prior to encystation is reported for the holozoan amoeba *Capsaspora owczarzaki*^23^, while the unicellular alga *Chlamydomonas reinhardtii* evolved multicellular structures when experimentally exposed for many generations to predation by ciliates^24^.

The quest to unravel ecological conditions in the distant past that caused transitions to multicellularity will most likely never yield fully conclusive answers. However, because these conditions will also have shaped the molecular mechanisms that regulate the intercellular interactions within the evolving multicellular organism, it is important to investigate and eventually include them in a comprehensive understanding of the physiology and behaviour of the extant multicellular forms.

## Methods

### Spore and cyst survival

For spores, *Dictyostelium* species were cultured in association with *Klebsiella aerogenes* on nutrient agar until fruiting bodies had formed. Spores were harvested in sterile PB (10 mM Na/K phosphate, pH 6.5) by rinsing fruiting bodies deposited on a 22 µm strainer with PB and collecting the eluate. Cysts were obtained by incubating cells for 3–5 days in 250 mM sorbitol in PB until fully encysted. Both cysts and spores were incubated for 1 min with 0.1% Triton to lyse any unencapsulated amoebas, washed twice with water and resuspended to 10^4^ cells/ml. Aliquots of 150 µl were dispensed in microfuge tubes and 10 tubes each were stored at 22 °C, 4 °C, and −20 °C. Additionally 15 µl of spores or cysts, re-suspended to 10^5^ cells/ml in water, were freeze-dried in microfuge tubes, which were afterwards sealed with parafilm and stored at −20 °C. After 0, 1, 7, 30, 91, 182 and 365 days of storage, freeze-dried samples were reconstituted with 150 µl water and triplicate 10 µl aliquots (containing 100 cells) of these and all other samples were mixed with 1.2 ml *K. aerogenes* suspension and distributed over 13 cm diameter nutrient agar plates. Plates were incubated at 22 °C until plaques were well established, which were subsequently counted. For later time points, aliquot size was increased to 20, 30 or 40 µl as needed to allow sufficient plaques to emerge for reliable counting.

### Transmission electron microscopy

*Dictyostelium* species were grown in association with *Klebsiella aerogenes* on 1/5^th^ SM agar for robust species and either LP, 1/3^rd^ LP or non-nutrient agar plates with charcoal for delicate or very delicate species, respectively^8^, and left to develop into fruiting bodies after bacteria were cleared. Mature spores were harvested from 4 day-old fruiting bodies. Cysts were induced by incubating cells, freshly harvested from growth plates, for 3–5 days in darkness submerged in PB or 250 mM sorbitol in PB, depending on the species^8^. Cysts were harvested when fully encysted, as established by Calcofluor staining of the cellulose wall. Spores and cysts were pelleted and fixed by submersion in 0.83% glutaraldehyde, 0.67% OsO4, 2.5% sucrose in 0.1 M sodium cacodylate pH 7.4 as described previously^25^. Dehydration, embedding, and sectioning used conventional techniques. Images were captured on Ditabis imaging plates or a SIS Megaview III camera using a JEOL 1200EX electron microscope. For each species, 10 images of complete spore- and cyst sections where collected at magnifications between 10 and 20 K, as well as close-ups of regions of the cell wall at 60 K. Sections were selected that were not obviously oblique, included a fair-sized nucleus and went through spores longitudinally, to ensure that they passed approximately through the centre of the cell.

### Morphometry

Electron micrographs were converted to 8-bit format and all quantitative features were measured with the line and area functions of ImageJ^26^. Magnifications were corrected using the embedded scale bar. Electron density was recorded as grey scale within the area of measurement of unedited images in Adobe Photoshop CS5 (www.adobe.com/products/photoshop.html). The following features were measured: The total width of the spore and cyst wall and of its constituent layers and the gray scale of the layers at 20 positions each of 10 cells. We further measured the number and diameter of spore and cyst granules, the number and total area of membrane-bound vesicles and the total cross-section area of cyst and spores, exclusive of the wall, over 10 cells each. The latter values were used to calculate the proportional contribution of granules and vesicles to the cross-section area. For qualitative features such as the grouping and location of granules and vesicles, the presence and distribution of heterochromatin in the nuclei and the presence of unusual features, the fraction of full cell sections that displayed the feature was scored. For mitochondrion associated features, such as a smooth or indented outline, closed versus open cristae and the presence and absence of a full fringe of ribosomes the fraction of mitochondria per cell that displayed the feature was determined. All measurements and calculations are listed in Supplemental files Data2_Walls.xlxs and Data3_Organelles.xlxs.

### Divergence time estimation

A concatenated alignment of 240 genes across 85 obazoan and amoebozoan species was constructed as described previously^27^. The alignment was trimmed from 99198 to 78464 positions by removing sections that were not unambiguously aligned or contained gaps over >50% of sequences. A phylogenetic tree was constructed using IQ tree (Fig. S14A), under LG + Г_4_ + C60 + PMSF with 100 bootstrap replicates. The topology of this tree was used to perform divergence time estimation using the program MCMCTree in the software package PAML v. 4.9^28^. The fossil calibration points in the metazoan, fungal and amoebozoan clades are listed in Table S4. The alignment was analysed as a single partition under the LG + Γ amino acid substitution model. The evolutionary rates were assumed to vary independently among lineages (the independent rates model) sampled from log-normal distributions^29^. The prior on the mean rate was set to be a gamma distribution with the shape parameter 2 and the scale parameter 20, and the prior on rate variability was set to be a gamma distribution with the shape parameter 1 and the scale parameter 10. The fossil calibrations were represented as uniform distributions with soft bounds, with 2.5% probability above or below maximum or minimum constraints, respectively. The approximate method of likelihood calculations in MCMCtree^30^ was used to calculate the approximate likelihoods in the divergence time analyses. Two MCMC chains were run for 2,500,000 iterations, sampled every 50 iterations, with a burn-in of 500,000 iterations. Convergence was confirmed by comparing the posterior means of the samples from two runs.

## Supplementary information

Supplementary spreadsheet Data1_Sporefitness.xlsx.

Supplementary spreadsheet Data2_Walls.xlsx.

Supplementary spreadsheet Data3_Organelles.xlsx.

Supplementary spreadsheet Data4_Correlations.xlsx.

Supplementary spreadsheet Data5_Ecology.xlsx.

Supplementary figures and tables.
