# Supplementary material for: Cold climate adaptation is a plausible cause for evolution of multicellular sporulation in Dictyostelia
Source: Sci Rep. 2020 May 29;10:8797. doi: 10.1038/s41598-020-65709-3 (PMC7260361; doi:10.1038/s41598-020-65709-3)
Supplement: Supplementary file 6 — Supplementary figures and tables. [file 41598_2020_65709_MOESM6_ESM.pdf]

Cold climate adaptation is a plausible cause for evolution of multicellular sporulation in Dictyostelia

Hajara M. Lawal[1], Christina Schilde[1], Koryu Kin[1], Matthew W. Brown[3], John James[1], Alan R Prescott[2] and Pauline Schaap[1]*

[1]School of Life Sciences, University of Dundee, Dundee DD1 5EH, UK
[2]Dundee Imaging Facility, University of Dundee, Dundee DD1 5EH, UK
[3]Department of Biological Sciences, Mississippi State University, Mississippi State, MS, USA

SUPPLEMENTARY INFORMATION

Contents

Dictyostelium species used in this study 2
Spore and cyst fitness 3
Figure S1. Spore survival curves 3
Figure S2. Comprehensive representation of spore/cyst fitness during long term storage. 4
Spore and cyst ultrastructure 5
Figure S3. Group 1 spores. 6
Figure S4. Group 2 spores 7
Figure S5. Group 3 spores. 8
Figure S6. Group 4 spores 10
Figure S7. Cyst ultrastructure. 11
Table S2. Species-specific morphological features 12
Quantification of ultrastructural features 14
Figure S8. Spore and cyst walls. 14
Figure S9. Organelles - Granules 16
Figure S10. Organelles - Vesicles 18
Figure S11. Organelles – Mitochondria 19
Figure S12. Cell and nuclear features 20
Correlations between spore/cyst fitness and morphology 21
Table S3. Correlation matrix. 21
Distribution of Dictyostelia across climate zones. 22
Figure S13. Global distribution of Dictyostelia and optimal culture temperatures. 22
Divergence time estimation 23
Figure S14. Divergence time estimation. 23
Table S4: List of fossil calibration points. 24
Table S5. Divergence time estimates at different prior settings 24
Supplemental References 24

Dictyostelium species used in this study

	Given species name	Strain	New classification
Group 1	*Dictyostelium deminutivum*	MexM19A	*Cavenderia deminutivum*
	Dictyostelium exiguum	TNS-C-199	*Cavenderia exigua*
	Dictyostelium parivsporum	OS126	*Cavenderia parivspora*
	Dictyostelium mexicanum	MexTF4B1	*Cavenderia mexicana*
	Dictyostelium medusoides	OH592	*Cavenderia medusoides*
	Dictyostelium myxobasis	NT2A	*Cavenderia myxobasis*
	Dictyostelium fasciculatum	SH3	*Cavenderia fasciculata*
Group 2	*Dictyostelium polycarpum*	OhioWilds	*Synstelium polycarpum*
	Acytostelium ellipticum	AE2	*Rostrostelium ellipticum*
	Acytostelium subglobosum	LB1	*Acytostelium subglobosum*
	Acytostelium leptosomum	FG12A	*Acytostelium leptosomum*
	Dictyostelium oculare	DB4B	*Heterostelium oculare*
	Polysphondylium arachnoideum	YA1	*Heterostelium arachnoideum*
	Polysphondylium equisetoides	B75B	*Heterostelium equisetoides*
	Polysphondylium filamentosum	SU1	*Heterostelium filamentosum*
	Polysphondylium pseudocandidum	TNS-C-91	*Heterostelium pseudocandidum*
	Polysphondylium pallidum	PN500	*Heterostelium album*
intermediate	*Dictyostelium polycephalum*	MY1-1	*Coremiostelium polycephalum*
Group 3	*Dictyostelium caveatum*	WS695	*Speleostelium caveatum*
	Dictyostelium lacteum	TK	*Tieghemostelium lacteum*
	Dictyostelium tenue	Pan52	*Raperostelium tenue*
	Dictyostelium monochasioides	HAG653	*Raperostelium monochasioides*
	Dictyostelium tenue	PJ6	*Raperostelium tenue*
	Dictyostelium lavandulum	B15	*Hagiwaraea lavandula*
	Dictyostelium coeruleostipes	CRLC53B	*Hagiwaraea coeruleostipes*
intermediate	*Polysphondylium violaceum*	QSvi11	*Polysphondylium violaceum*
Group 4	*Dictyostelium purpureum*	QSDP1	*Dictyostelium purpureum*
	Dictyostelium austroandinum	Moreno 7	*Dictyostelium austroandinum*
	Dictyostelium capitatum	91HO50	*Dictyostelium capitatum*
	Dictyostelium mucoroides	S28b	*Dictyostelium mucoroides*
	Dictyostelium discoideum	NC4	*Dictyostelium discoideum*
	Dictyostelium robustum	TNS-C-219	*Dictyostelium robustum*
	Dictyostelium sphaerocephalum	GR11	*Dictyostelium sphaerocephalum*
	Dictyostelium clavatum	TNS-C-220	*Dictyostelium clavatum*

Species are phylogenetically ordered within their taxon group. We use the older given species names in this work instead of the novel classification (4th column) based on the SSU rDNA phylogeny [1,2], which did not resolve several genera and may need to be revised in the near future. Species are colour-coded to reflect the major taxon group of which they are part. Species in black text belong to minor intermediate groupings.

Spore and cyst fitness

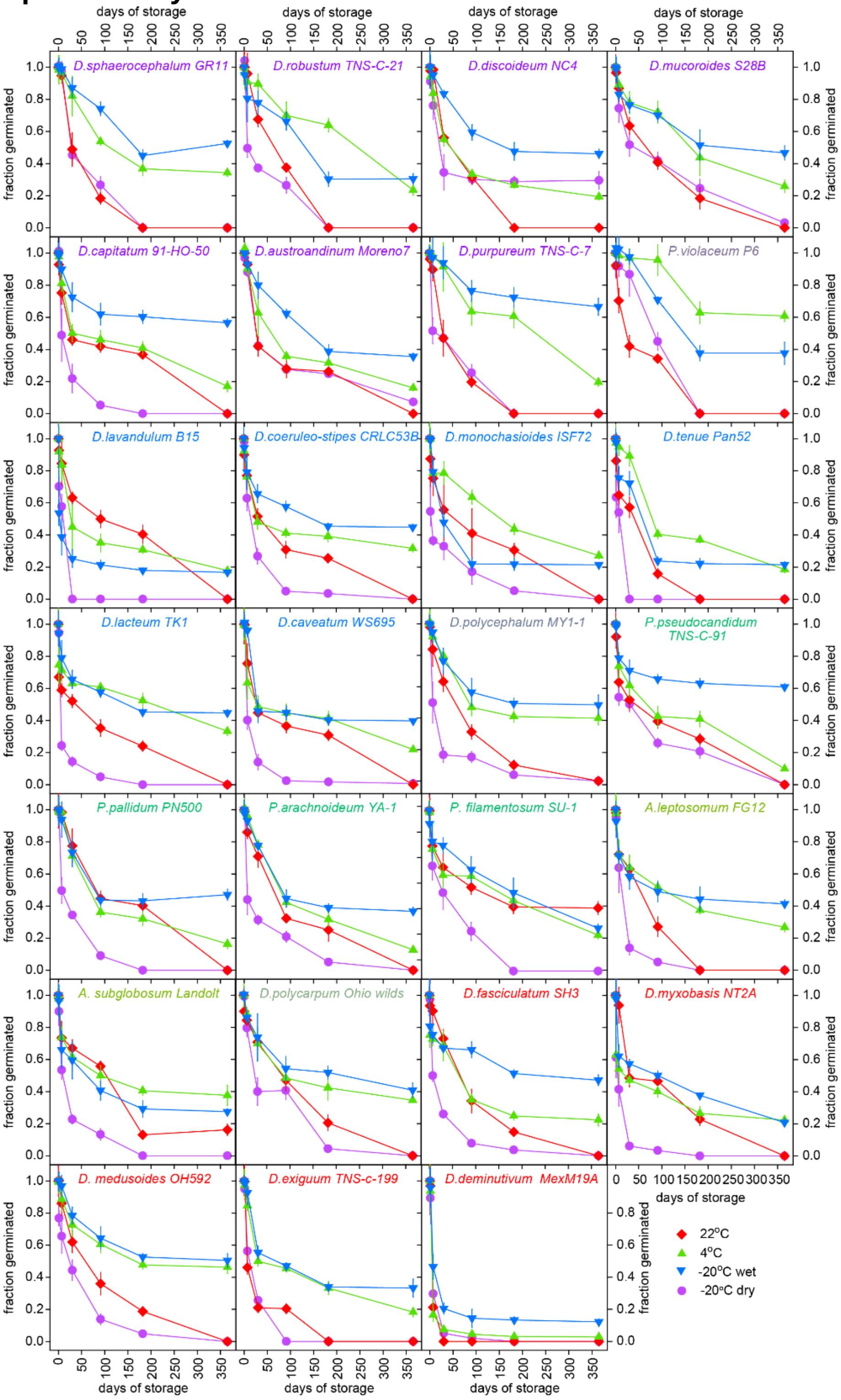

Figure S1. Spore survival curves

Mature spores from 27 species were stored as 150 µl aliquots of 10^4 cells/ml at 22°C, 4°C, and -20°C, or freeze dried as 15 µl aliquots of 10^5 cells/ml in water and stored dry at -20°C. After 0, 1, 7, 30, 91, 182 and 365 days of storage, freeze-dried cells were resuspended in 150 µl water and triplicate 10-40 µl aliquots of all stored samples were mixed with *K. aerogenes* suspension and distributed over large nutrient agar plates. After incubation at 22°C for several days, emerging plaques were counted. Counts were recalculated as fraction of plaques emerging from plating at t=0 days without treatment (See Data1_sporefitness.xlxs, sheet 2). Means and SD of triplicate measurements.

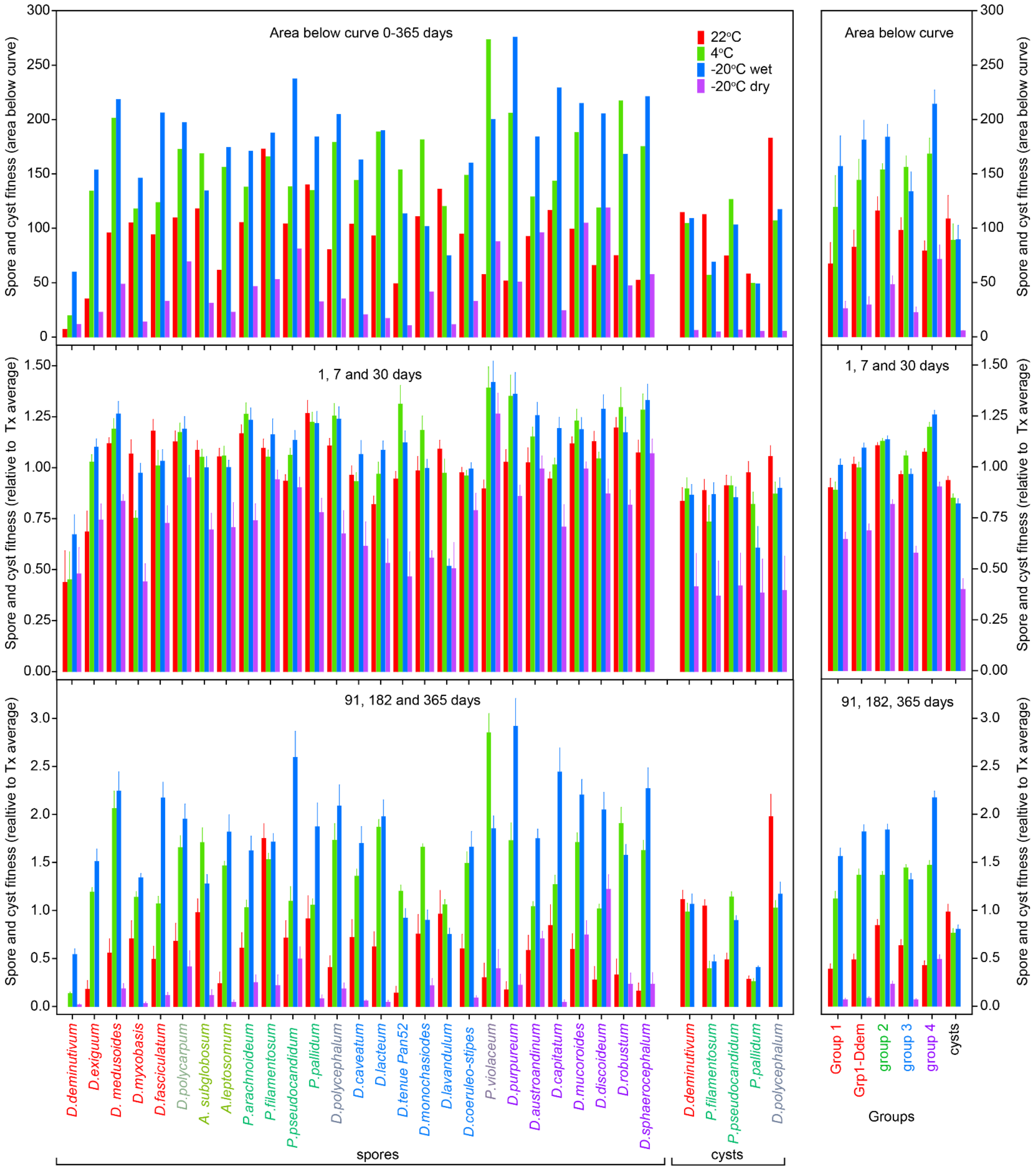

Figure S2. Comprehensive representation of spore/cyst fitness during long term storage.
A. *Area below curve.* To reduce the survival curves shown in figures 2A and S1 to a single value, the area below each curve was calculated using the trapeziodal rule and plotted per species for all storage regimens. A value of 365 (days × surviving fraction) equals 100% survival.

B/C. *Averaged relative survival per time point*. To retain the variance in the data set, spore and cyst fitness (Fx) values were expressed per time point (Tx) as fraction of the averaged survival at Tx over spores of all species over all storage conditions. The mean and SE of Fx values over 1,7 and 30 days (B) and 91, 182 and 365 days (C) were calculated next and are shown per species and per taxon group (See Data1_Sporefitness.xlsx, sheets 3-6).

Spore and cyst ultrastructure

To understand why spores survive long term storage better than cysts and why group 4 spores are more frost resistant than others, we examined ultrastructural differences between spores and cysts. Spore ultrastructure was investigated in at least six species from each of the major taxon groups and one species each for the minor groups, while cyst ultrastructure was studied for those species that readily formed cysts. Spores across taxon groups have many ultrastructural features in common. Spore cytoplasm is highly condensed with ribosomes and organelles packed closely together. This condensation obscures resolution of finer ultrastructural detail and features like endoplasmic reticulum and Golgi cisternae can generally not be distinguished. Membrane-bound vesicles with or without inclusions are common, and electron-transparent granules without membranes are present, mostly at the poles in groups 1-3. *Dictyostelium* nuclei have no identifiable single nucleosome, but can harbour distinct patches or more dispersed darker heterochromatin. The spore wall typically consists of two narrow dense layers that flank a broader more transparent layer. In *D. discoideum*, the latter was shown to consist mostly of cellulose fibrils, while the dense layers are more (glyco)protein-rich [3]. The spore surface can vary from smooth to undulating or sharply ridged.

Spores from all investigated species per group are shown in figures S3-S6, while cysts are shown in figure S7. Apart from *D. deminutivum* with a two-layered wall, group 1 spores have the typical three-layered spore wall and are further characterized by their tightly clustered polar granules embedded in smooth dark material. They usually show many grouped vesicles and round to oval mitochondria with visible cristae (Fig. S3). Characteristic for most group 2 spores are the loosely grouped polar granules, which can be surrounded by a double layer of ribosomes, likely rough endoplasmic reticulum (RER), but not by smooth dense material as in groups 1, 3 and 4. RER flanked granules are however also present in spores of the other groups and in cysts. Mitochondria are generally smooth-surfaced with well-defined cristae. Spores of many group 2 species also have a distinctive opaque sub-membrane cortex and many vesicles (Fig. S4). Group 3 spores are characterized by dense cytoplasm and relatively thin walls. Spore granules are clustered at the poles and are mostly surrounded by denser smooth material. Mitochondria are often broadly or more finely lobed (Fig. S5). Group 4 spores stand out by having spore granules that are not distinctly polar, but dispersed throughout the cell. Most mitochondria are finely lobed (crenate), with almost invisible cristae and, unique for this group, they are fringed with ribosomes (Fig. S6).

Cysts contain semi-transparent granules that are similar to the spore polar granules and many vesicles. However, their wall architectures are more variable (Fig. S7). *D. deminutivum* cysts in group 1 have a narrow inner transparent layer and even thinner outer slightly denser layer. Group 2 cysts have a one or two-layered wall, the latter with the inner being more opaque and the outer layer loosely fibrous. *D. polycephalum*, an outgroup species to both groups 3 and 4 has a relatively wide wall that is less structured than the spore wall. See the legends to figures S3-S6 for more detailed descriptions of spores and cysts for each species and table S2 for species-specific features.

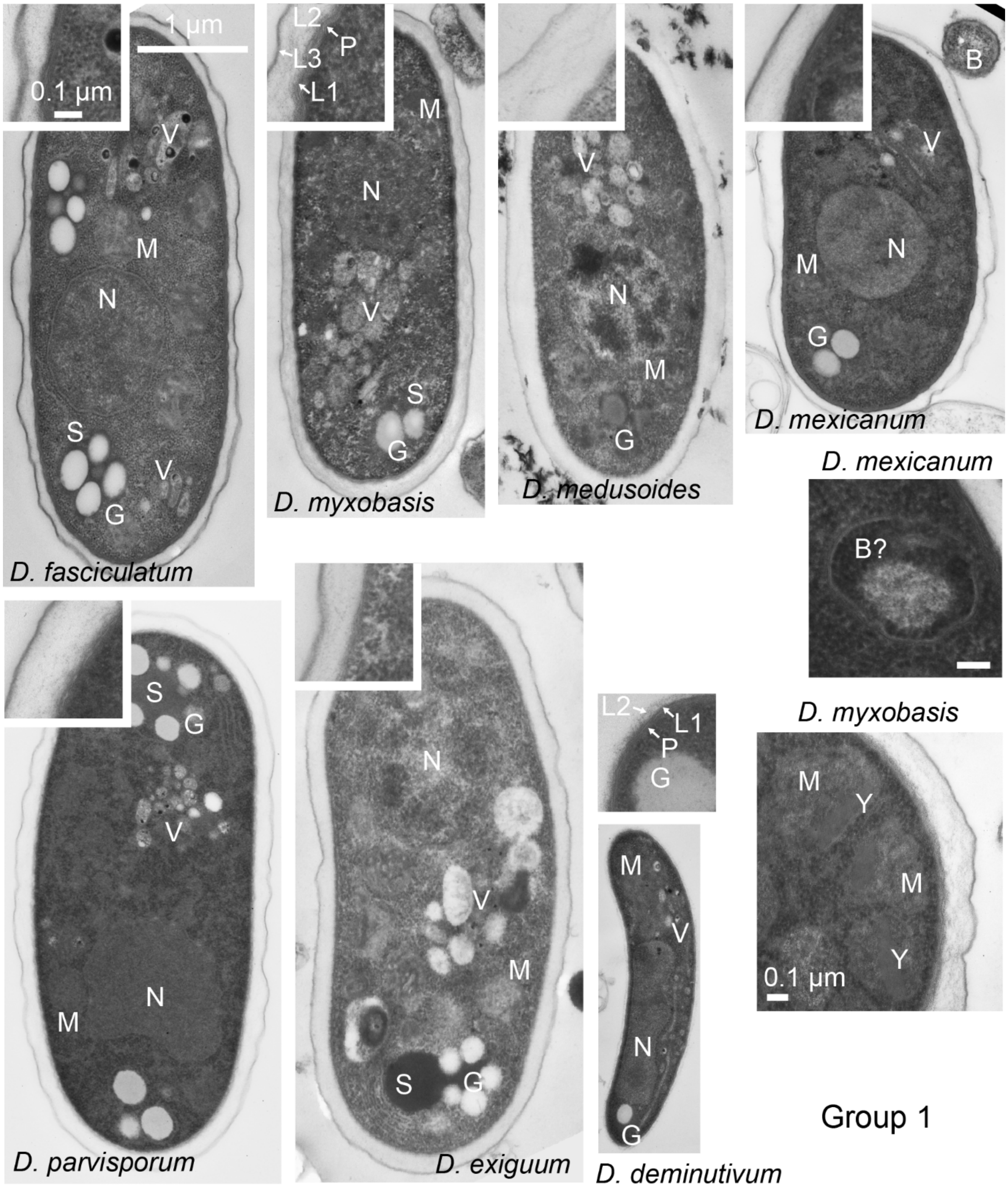

Figure S3. Group 1 spores.

D. fasciculatum spores show the characteristic three-layered spore wall and condensed cytoplasm. Spore granules (G) are closely grouped at or below the poles, often embedded in smooth darker material (S) and the spore wall varies locally in width. The heterochromatin is mostly dispersed throughout the nucleus (N) and mitochrondria (M) show visible tubular cristae. Many grouped vesicles (V) are present at different locations. The *D.myxobasis* spores are similar to those of *D. fasciculatum*, except that the middle layer (L2) of the spore wall is more fibrous, the number of polar granules is relatively low and crystals (Y) are observed within mitochondria. The *D.medusoides, D. mexicanum, D. parvisporum* and *D. exiguum* spores resemble those of *D. fasciculatum,* but have more homogeneous wall width, while *D. mexicanum* spores contain bacteria enclosed in vesicles (B?). The *D. deminutivum* spores are very small with a thin 2-layered wall consisting of a thin dense inner and loosely fibrous outer layer. There are both large vesicles, stretched out along the length of the spore and smaller grouped

vesicles. There is generally only one spore granule per pole. The overview or inset images are shown at the same magnification. Bar in overview: 1 µm; bar in inset: 0.1 µm. P: plasma membrane, L1, L2, L3: inner, middle and outer layers of spore wall.

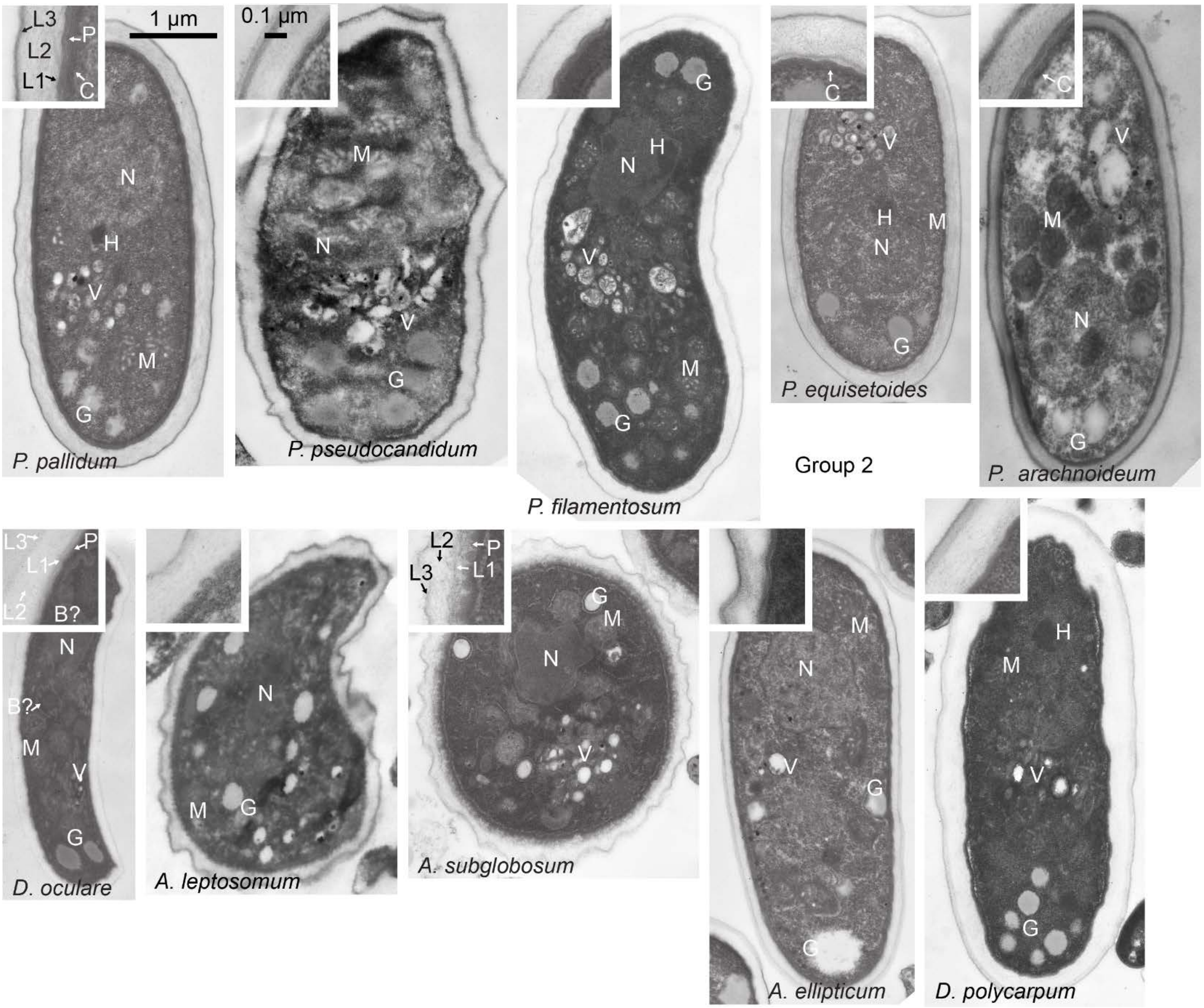

Figure S4. Group 2 spores

P. pallidum spores have a fairly wide three-layered walls and a distinctive dark cortex (C) below the plasma membrane (P). Spore granules are loosely grouped at the poles. There are many grouped and dispersed vesicles and mitochrondria show distinctive cristae. *P. pseudocandidum*, *P. filamentosum*, *P. equisetoides* and *P. arachnoideum* spores are similar to those of *P. pallidum*, except that the cytoplasm of *P. arachnoideum* spores shows typical empty patches. *D. oculare* spores are small with a three-layered wall that sometimes forms acute angles at the poles. Mitochondria show distinct cristae and some inclusions. There are many small vesicles, mostly grouped and present throughout cell. Most spores also have some larger highly indented vesicles with dense granular content, resembling the contents of *K. aerogenes* food bacteria (B?). One or two large granules are present at the poles, giving the impression of eyes, to which this species owes its name.

The plasma membrane and underlying cortex of *A. leptosomum* and *A. subglobosum* spores are intensely scallopped. Spore granules are dispersed throughout the cells. The elliptical spores of *A. ellipticum* have a relatively narrow wall and display spore granules of variable size. Mitochondria show distinctive cristae. *D.*

polycarpum spores have a thick spore wall and loosely grouped polar granules. The plasma membrane and underlying cortex is in places discontinuous with the underlying cytoplasm, which may be due to shrinkage during fixation. Bar in overview = 1 µm; bar in inset: 0.1 µm.

–

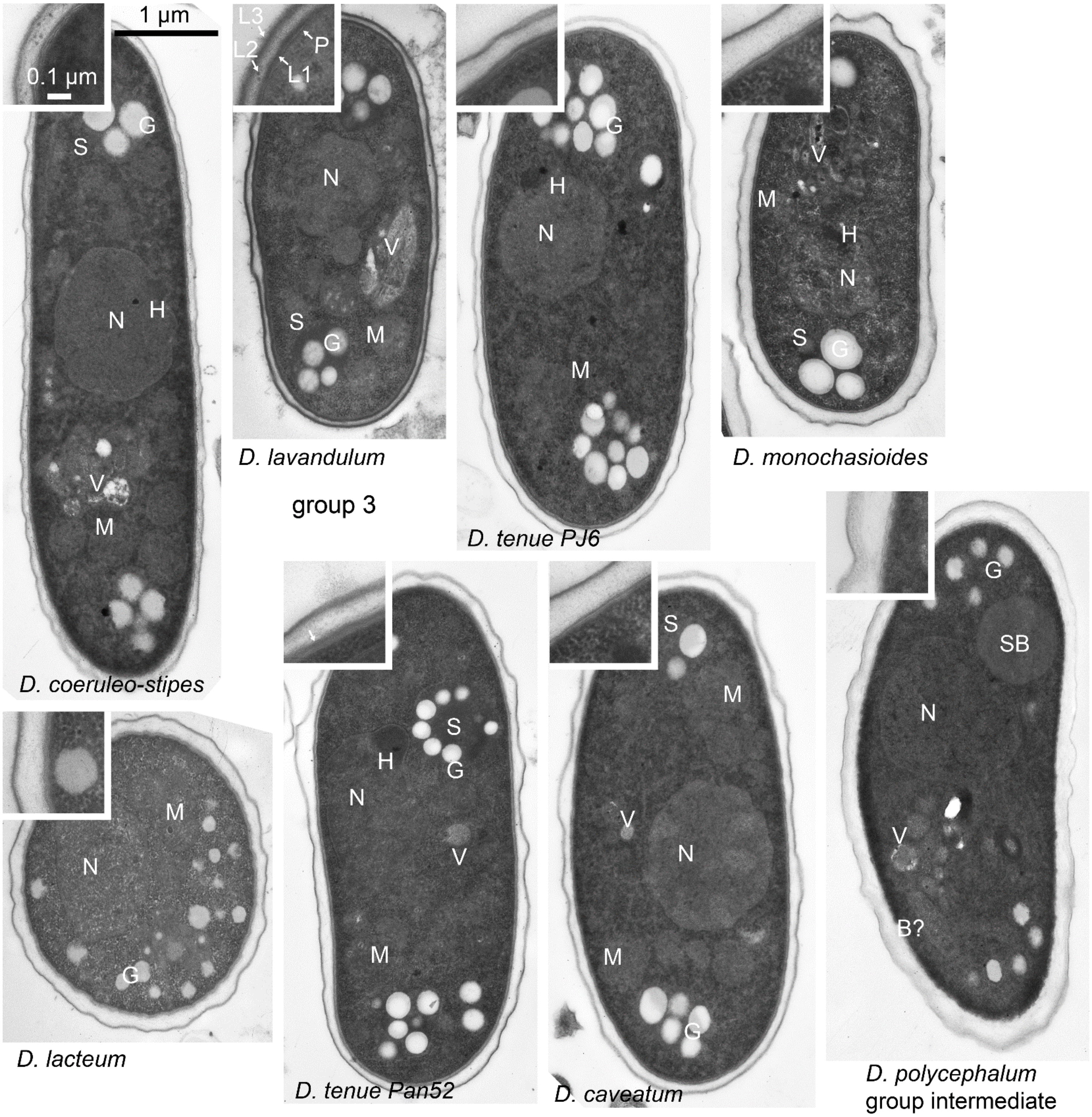

Figure S5. Group 3 spores.

D. coeruleo-stipes spores are long and narrow. The spore wall is relatively thin, but the middle layer is rather fibrous with some dense spots. Spore granules are grouped together at or just below poles, mostly embedded in darker smooth material. Mitochondria are often broadly lobed. The polar granule content of the thin-walled

D.lavandulum spores is somewhat heterogeneous and the granules are often embedded in smooth dark material. Mitochondria show mostly open cristae. *D. tenue Pan53, D. monochasioides* spores and D. tenue PJ6 are similar to those of *D. lavandalum*, with *D. monochasioides* spores having thicker walls. *D. lacteum* has globose spores with the typical three-layered spore wall. There are many spore granules, loosely grouped or dispersed throughout the spore. *D. caveatum* spores are again similar to those of *D. lavandulum*.

D. polycephalum is the closest outgroup to groups 3 and 4. Spore granules are mostly localized at the poles, but more dispersed than those of group 3 spores. Most spores contain one or several bodies that are very similar to intact *K. aerogenes* bacteria (B?) as well as a large smooth body (SB) of intermediate electron opacity. Bar in overview = 1 µm; bar in inset: 0.1 µm.

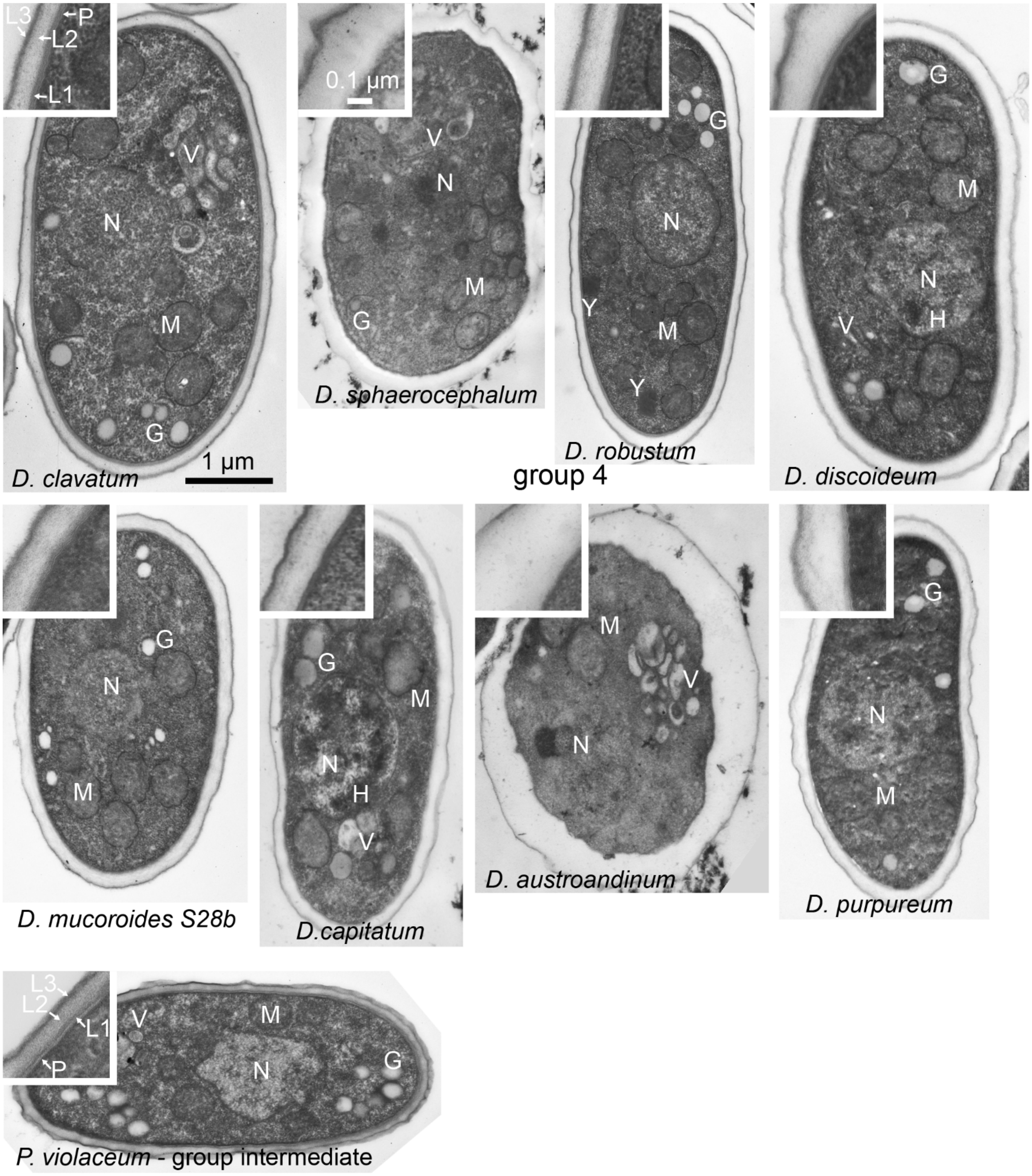

Figure S6. Group 4 spores

D. clavatum spores show the typical three-layered spore wall and condensed cell content. Mitochondria (M) are both smooth-surfaced or distinctly crenate with poorly visible cristae. Unique for group 4, mitochondria are fringed by ribosomes. Several semi-transparent granules (G) are present at the poles and throughout the cell. Vesicles are present, mostly perinuclear in groups, but also dispersed throughout the cell. *D. sphaerocephalum* spores are similar to *D. clavatum* spores, but have thicker walls and crystal-like inclusions (Y). Crenate mitochondria with closed cristae are more prominent in all other group 4 spores and crystals were observed in the cytosol of

all species except *D. austroandinum.* In *D. discoideum* amoebas, similar crystals are formed by an esterase protein [4]. *D. austroandinum* spores walls are very wide and those of *D. mucoroides* are more fibrous than others. The content of the *D. purpureum* spores is very condensed. The ultrastructure of the organelles is therefore poorly defined, but similar to that of *D. mucoroides* and the other group 4 species.

P. violaceum is a member of a small sister group to group 4. Its spore granules are more specifically localized at the poles and are sometimes embedded in darker smooth material. Mitochondria have open crista and are less frequently fringed by ribosomes than those of group 4 spores. The outer spore surface shows sharp peaks or ridges. Bar in overview: 1 µm; bar in inset: 0.1 µm.

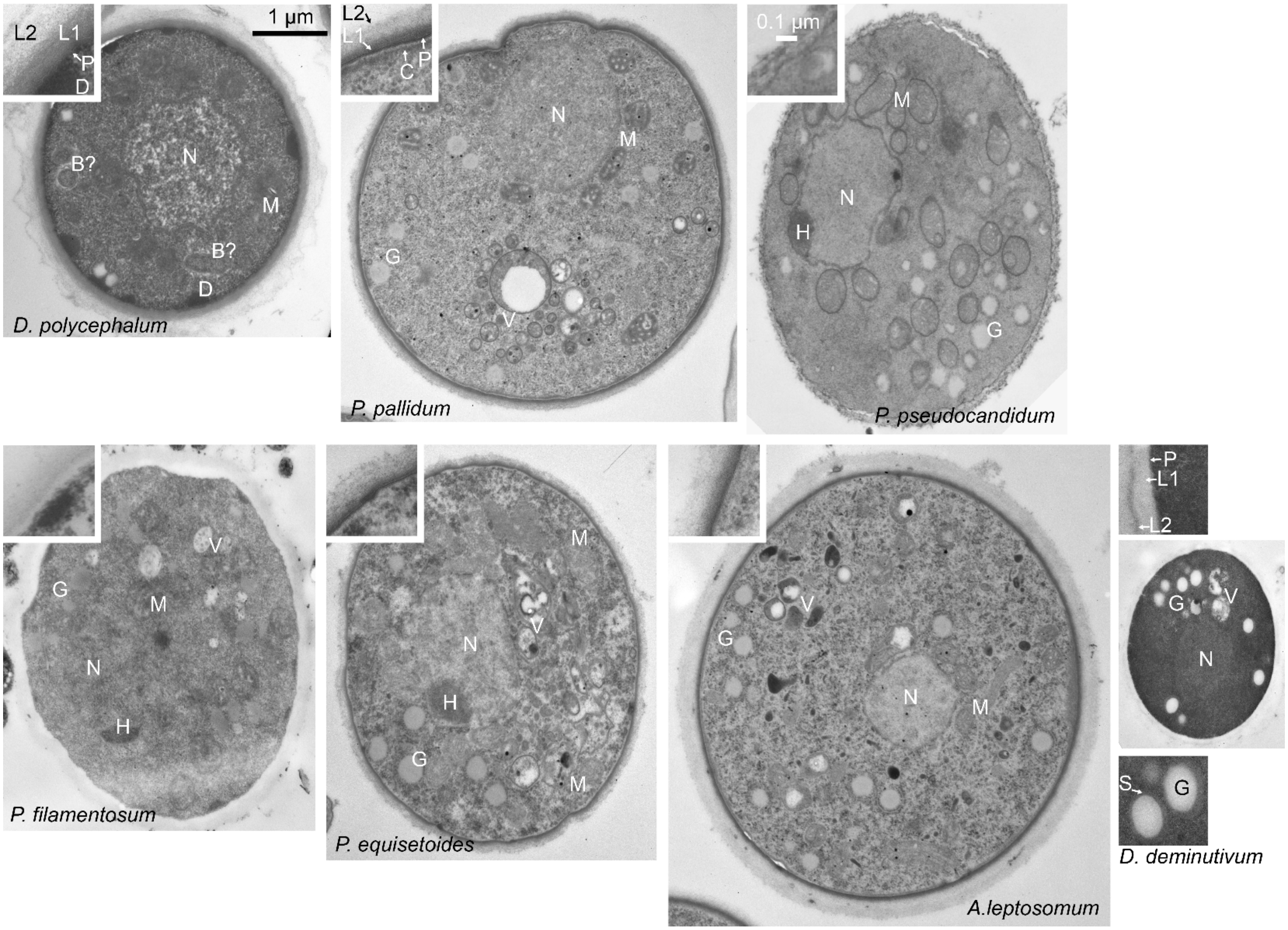

Figure S7. Cyst ultrastructure

D. polycephalum cysts show a fairly dense inner wall and a loosely fibrous outer wall of varying thickness. There is a somewhat denser top layer, which is however much less defined as the spore outer layer and therefore not considered as a third layer here. Mitochondria are round with distinct cristae and some inclusions. Granules are dispersed through the cell. Several hemispherical dark bodies are present underneath the plasma membrane and bacteria-like inclusions (B?), similar to those seen in *D. polycephalum* spores, are present in most cysts. The *P. pallidum* cyst walls consist of an inner layer of intermediate density and an outer loosely fibrous layer. The cyst content is less condensed than that of the spore. Mitochondria with well-defined cristae, sometimes with inclusions, are localized throughout the cell. Granules and vesicles of varying size are present throughout the cell. A narrow discontinuous cortex is visible below the plasma membrane. The ultrastructure of *P. pallidum* cysts has been reported previously [5] and is almost identical to that described here. *P. pseudocandidum* cysts show a single layered highly fibrous cell wall and opaque inner membranes. The *P. filamentosum* cysts show

similar hemispherical opaque patches below the plasma membrane as *D. polycephalum* cysts. The wall appears to be only single layered and is rather transparent. Similar to those of *P. pallidum, A. leptosomum* cysts walls show a narrow dark inner and wider more transparent outer layer. However, a cortex is not obvious. Mitochondria are round to oval with well-defined cristae and both granules and vesicles are present throughout the cytoplasm. The *P. equisetoides* cytoplasm contains typical "empty" patches, devoid of ribosomes. *D. deminutivum* cysts are, like the spores, very small with highly condensed contents. The cyst wall is insubstantial and consists of an inner translucent layer and narrow denser outer layer. Single and grouped vesicles are present and granules are dispersed throughout the cell, often enclosed in smooth darker material. Bar in overview: 1 µm; bar in inset: 0.1 µm.

Table S2. Species-specific morphological features

**Species**	**Feature A**	**fr.**	**Feature B**	**fr.**
D. deminutivum	highly flattened vesicles	0.3		
D.exiguum	granules embedded in smooth dark material	0.4		
D. parvisporum	granules embedded in smooth dark material	0.7		
D. mexicanum	vesicles with intact bacteria	0.3		
D. myxobasis	crystals in mitochondria	1.0	fibrous middle wall layer	1
D. fasciculatum	granules embedded in smooth dark material	0.5	undulating wall surface	1
D. polycarpum	corrugated inner wall layer	1	thick smooth wall	1
A. ellipticum	one large and several smaller opaque granules	0.7		
A. subglobosum	cortex intensely scallopped	1	wall very fibrous and corrugated	1
D. oculare	indented vesicles with bacteria	0.8		
P.arachnoideum	granules with double ring of ribosomes	0.4		
P. equisetoides	distinctive cortex, somewhat corrugated	1	fibrous middle wall layer	1
P. filamentosum	distinctive cortex	1		
P. pseudo-candidum	distinctive cortex	0.8		
P. pallidum	distinctive cortex	1		
D. polycephalum	large medium dark spot	0.3	bacteria, unclear whether in vesicles	0.7
D. caveatum	granules embedded in smooth dark material	0.5		
D. lacteum	outer wall finely tufted	1		
D. tenue Pan52	granules embedded in smooth dark material	0.6	undulating wall surface	1
D. monocha-siodes	granules embedded in smooth dark material	0.3		
D. tenue PJ6	undulating wall surface	1		
D. lavandulum	granules embedded in smooth dark material	0.7		
D. coeruleo-stipes	granules embedded in smooth dark material	1	dark spots in middle wall layer	1
P. violaceum	sharply ridged wall surface	1		
D. purpureum	crystal(s) in cytosol	0.3		
D.austroandinum	dense bands, often in stacks, in cytosol	0.4		
D. capitatum	crystal(s) in cytosol	0.3		
D. mucoroides	crystal(s) in cytosol	0.3	fibrous middle wall layer	1
D. discoideum	crystal(s) in cytosol	0.3		
D. robustum	granules embedded in smooth dark material	0.3	crystal(s) in cytosol	0.6
D. sphaero-cephalum	crystal(s) in cytosol	0.5		
D. clavatum	granules embedded in smooth dark material	0.2		

**cysts**				
D. deminutivum	granules in ring of smooth darker material	0.4	cytosol very dense	0.6
P. equisetoides	"empty" patches in cytosol	1		
P. filamentosum	hemispherical dark patches below plasmamembrane	0.8	Single layered cyst wall	1
P. pseudo-candidum	Interior membranes very opaque	1	single layered highly fibrous cell wall	1
P. pallidum	vesicles between plasmamembrane and wall	0.4	invagination of periphery	0.6
D. polycephalum	hemispherical dark patches below plasmamembrane	1	bacteria, unclear whether in vesicle	0.5

Listing of ultrastructural features found in only one or a few species, with the fraction (fr.) of cells that displayed the feature. Features that appeared multiple times have the same text color.

Quantification of ultrastructural features

To aid objective evaluation of differences between spores or cysts of individual species, we measured or enumerated ultrastructural features from 10 spores or cysts for each species. These features are the width of each layer of the spore and cyst wall, the location, grouping, number and diameter of granules per section and their fractional contribution to the cell cross-section area, the grouping, number and fractional cross-section area of membrane-bound vesicles per section, the distribution of heterochromatin and the morphology of mitochondria in each cell. Features that were only found in one or a few species are listed in Table S2.

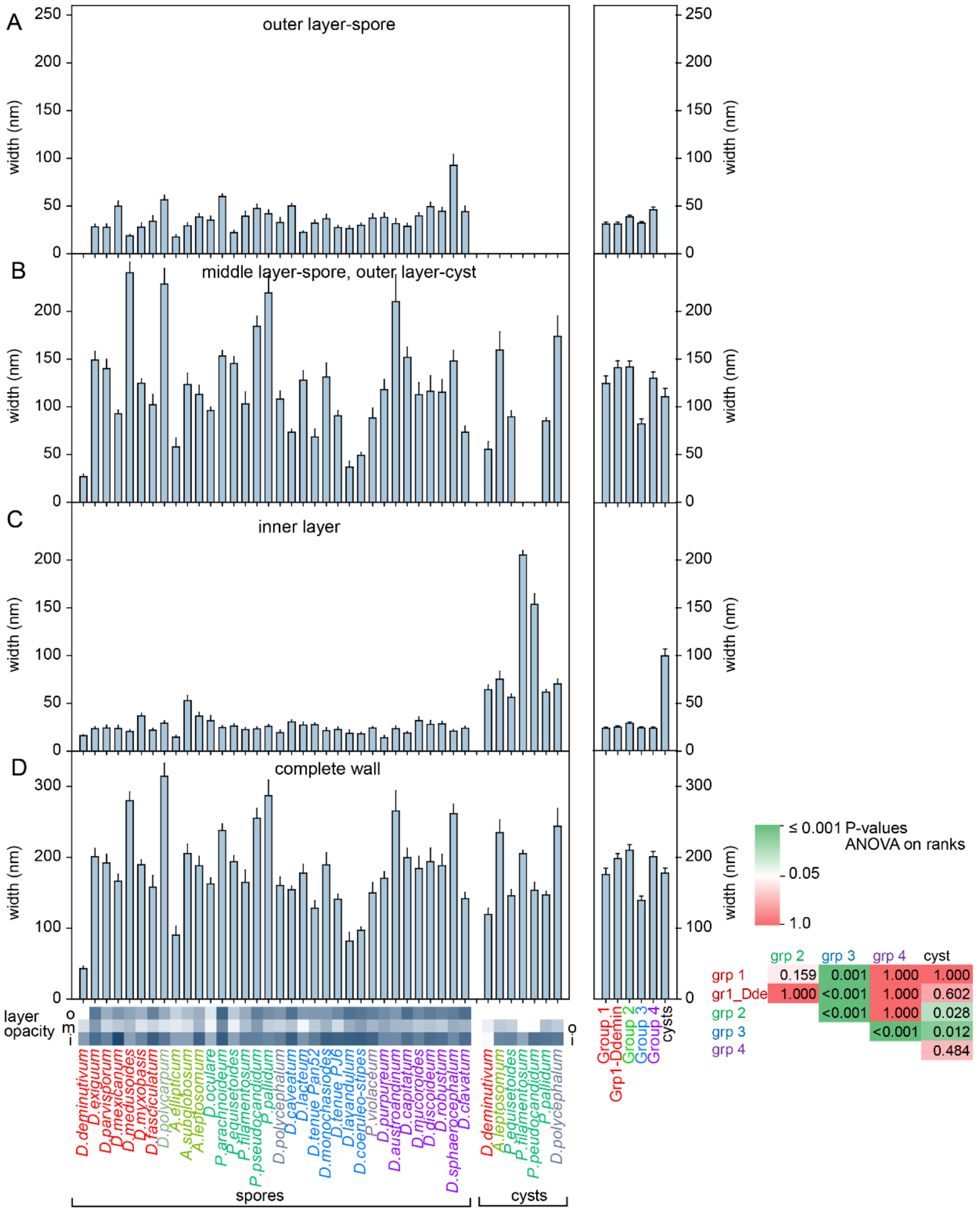

Figure S8. Spore and cyst walls.

The width of spore and cyst wall layers were measured at 20 positions across the periphery of TEM sections of 10 different cells and averaged per cell. These values were then averaged per species and displayed with standard error of the mean (SE) values (n=10) in the left panels. All per cell values were also averaged over entire taxon groups and shown with SE in the right panels. Because *D. deminutivum* with its very small spores

and narrow walls is a distinct outlier, the values for group 1 were also calculated without those for *D. deminutivum* (Grp1-Ddemin.). For complete wall width significant differences between groups (P<0.05) were tested using Analysis Of Variance (ANOVA) on ranks. P-values of the comparisons are shown in the colour coded matrix. All data and statistical analyses are listed in supplemental spreadsheet Data2_Walls.xlxs.

Within groups there is considerable variation in the width of the spore wall, which is largely due to variation in the central cellulosic layer. Measurements of the gray scale of the wall layers from EM images show that the middle layer is always the least electron opaque of the three layers. For all cysts, except *D. deminutivum,* the inner layer is the most opaque. Note that due to variation in section thickness and counter-staining, the gray scale values only provide a relative estimate of electron opacity. Across taxon groups, group 3 walls are significantly narrower than those of groups 2, 4 and 1.

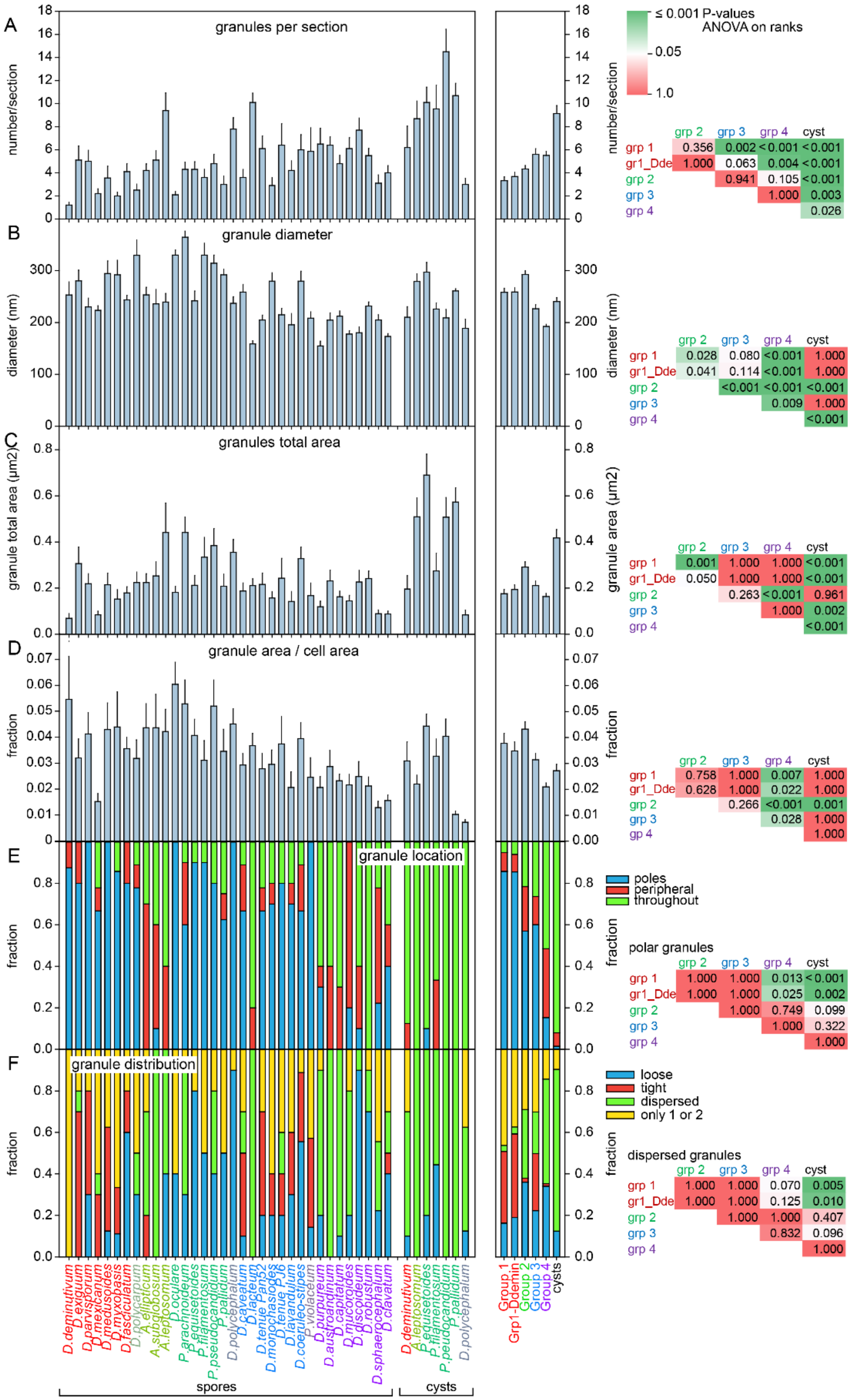

Figure S9. Organelles - Granules

Spore and cyst granules are defined as circular semi-transparent organelles that are not enclosed in membranes and usually reside at the poles of spores of groups 1 and 3. They are considered to be lipid droplets although this was never proven by (histo)chemical analysis. We determined the number (A) and average diameter (B) of granules per section over sections of 10 cells for each species as well as the total section area without the wall (Fig. S12A) and calculated the total (C) and relative area (E) occupied by granules per section. In addition we recorded the location (E) and distribution (F) of granules, the latter only when more than two

granules were present. The bar graphs (A-C) represent means and SE of quantitative features for individual species on the left panels and for taxon groups on the right panels. Qualitative features (E,F) are presented as stacked bars, where the length of each stack represents the fraction of cells that display the feature.

Cysts contained significantly more granules than spores (A), which were relatively large (B). However, due due to cysts being larger than spores, their proportional contribution to the section area was about the same (D). Spore granules were relative large in groups 1 and 2 and were significantly smaller in group 4 (B). Their proportional contribution to the cell area was also significantly smaller in group 4 (D). In both cysts and group 4 spores, granules were mostly distributed throughout the cell (E), while in spores from groups 1-3, granules were predominantly located at the poles. Group 2 granules tended to be more loosely grouped than those in groups 1 and 3 (E). Note that the low number of granules visible per section makes assessment of their grouping in TEM images less reliable than from whole spore observations under phase contrast [6]. All data, calculations and statistical analyses are listed in supplemental spreadsheet Data3_Organelles.xlxs.

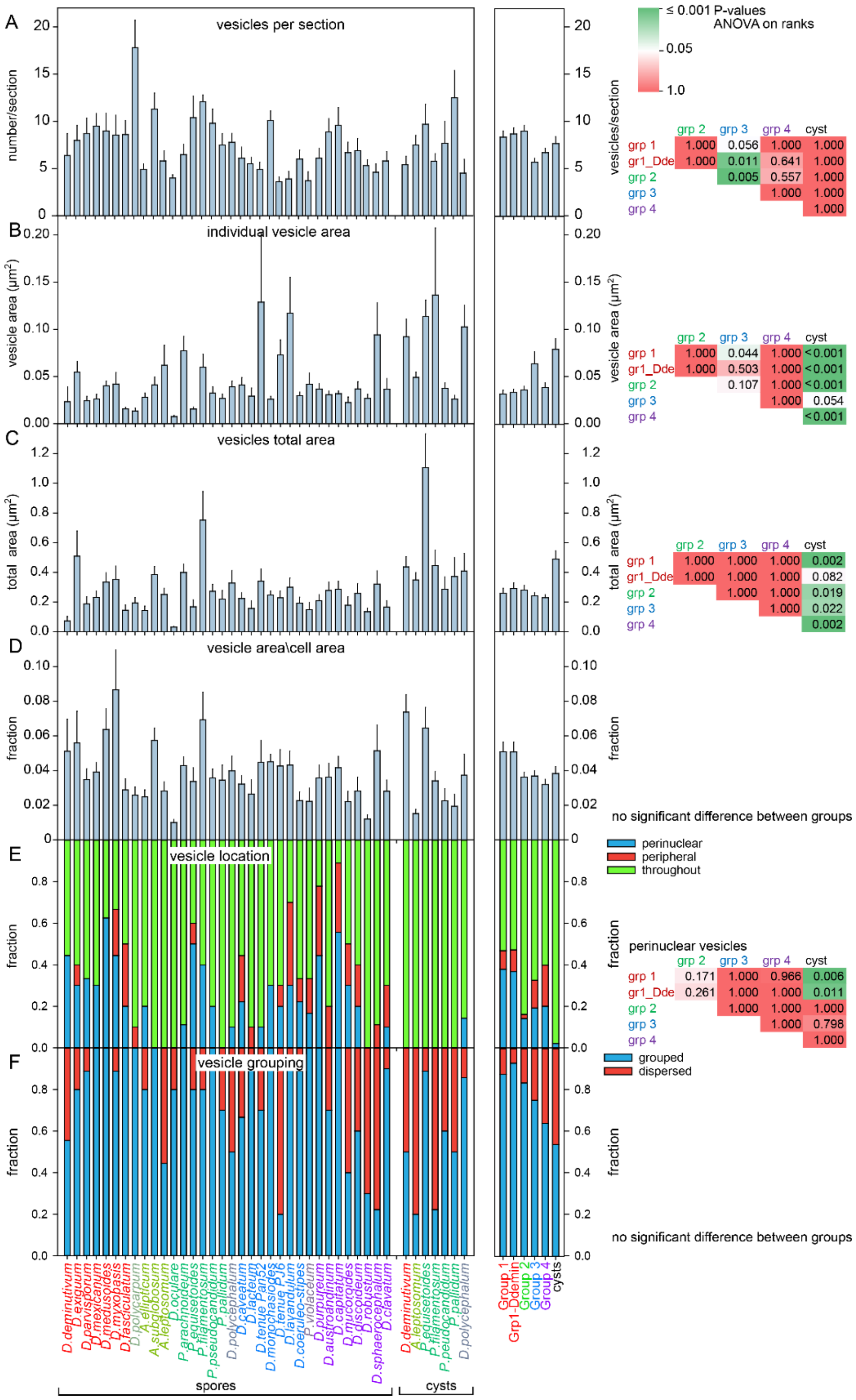

Figure S10. Organelles - Vesicles

Vesicles here comprise all membrane bounded organelles that are not mitochondria or the nucleus. Their numbers were counted per section (A) and their total area was determined by circling the circumference of individual or of tightly packed clusters of vesicles onscreen in ImageJ (C). Individual vesicle area was assessed by dividing total vesicle area per section by vesicle number (B). Relative vesicle areas (D) and the location (E) and distribution (F) of vesicles were determined as described for granules. Partially due to the large variation in vesicle abundance and size between sections and between species, none of the measured parameters showed significant differences between spores of different taxon groups. The relatively large cysts contained significantly larger but not more numerous vesicles than spores, but the overall contribution of vesicles to their cross section area was about the same.

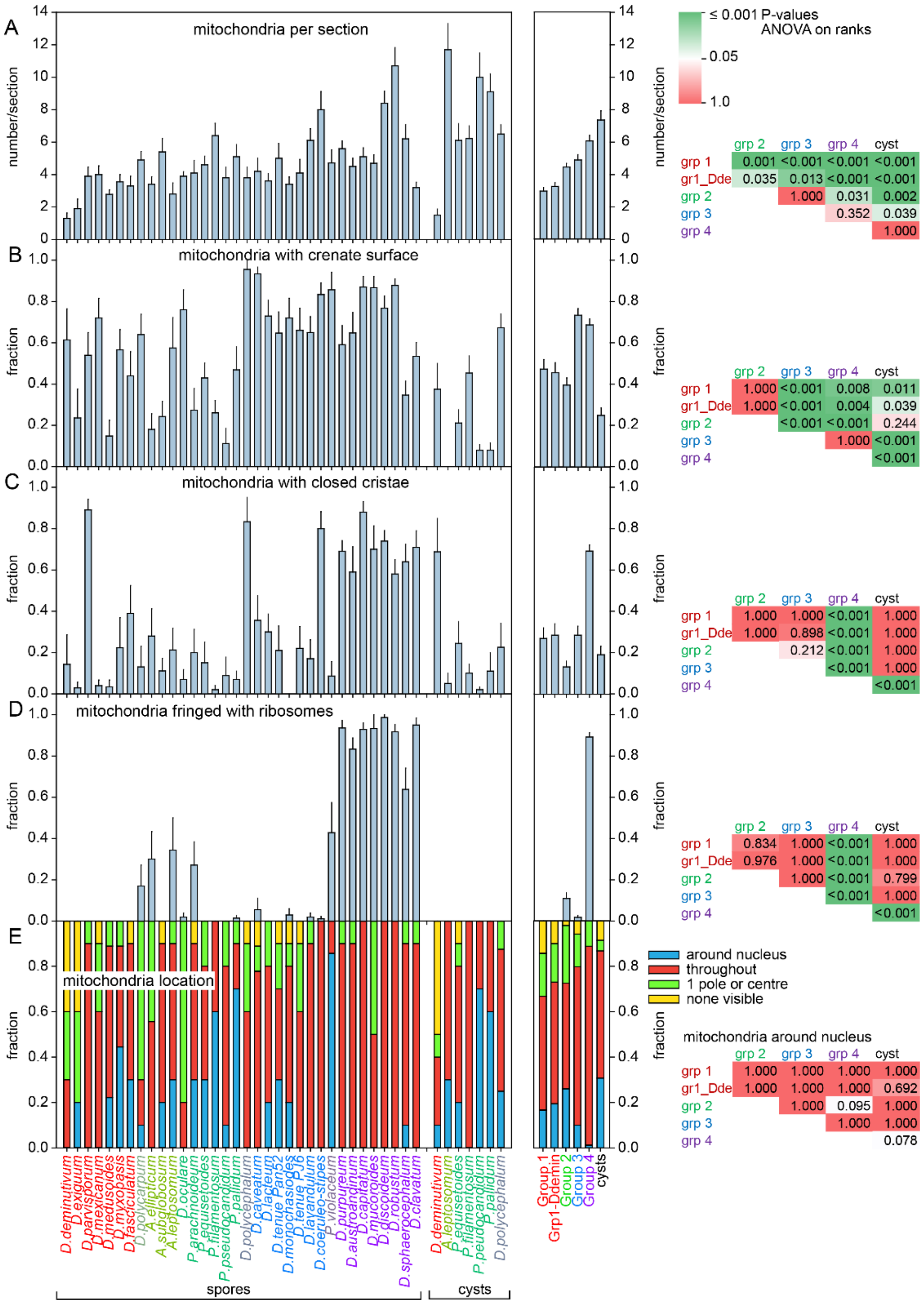

Figure S11. Organelles – Mitochondria

The outlines of the relatively opaque mitochondria could be difficult to distinguish in the equally opaque spore cytoplasm. We therefore did not attempt to measure their area, but counted their numbers per cell (A) and the fraction of mitochondria per cell that displayed indented (crenate) versus smooth outlines (B), closed versus open cristae (C) and presence versus absence of a border of ribosomes (D). These features appeared to show group-specific differences upon initial examination of EM images. We additionally recorded the distribution of mitochondria over the cell (E).

Mitochondria are most numerous in cysts followed by group 4 and groups 3, 2 and 1 in sequence, which may be related to cell size also decreasing in roughly that order (Fig. S12). Mitochondria with an indented surface are significantly most abundant in groups 3 and 4 than in the other groups or in cysts, while mitochondria with closed cristae and a ribosome fringe are particularly prominent in group 4. The location of mitochondria does not differ significantly between groups.

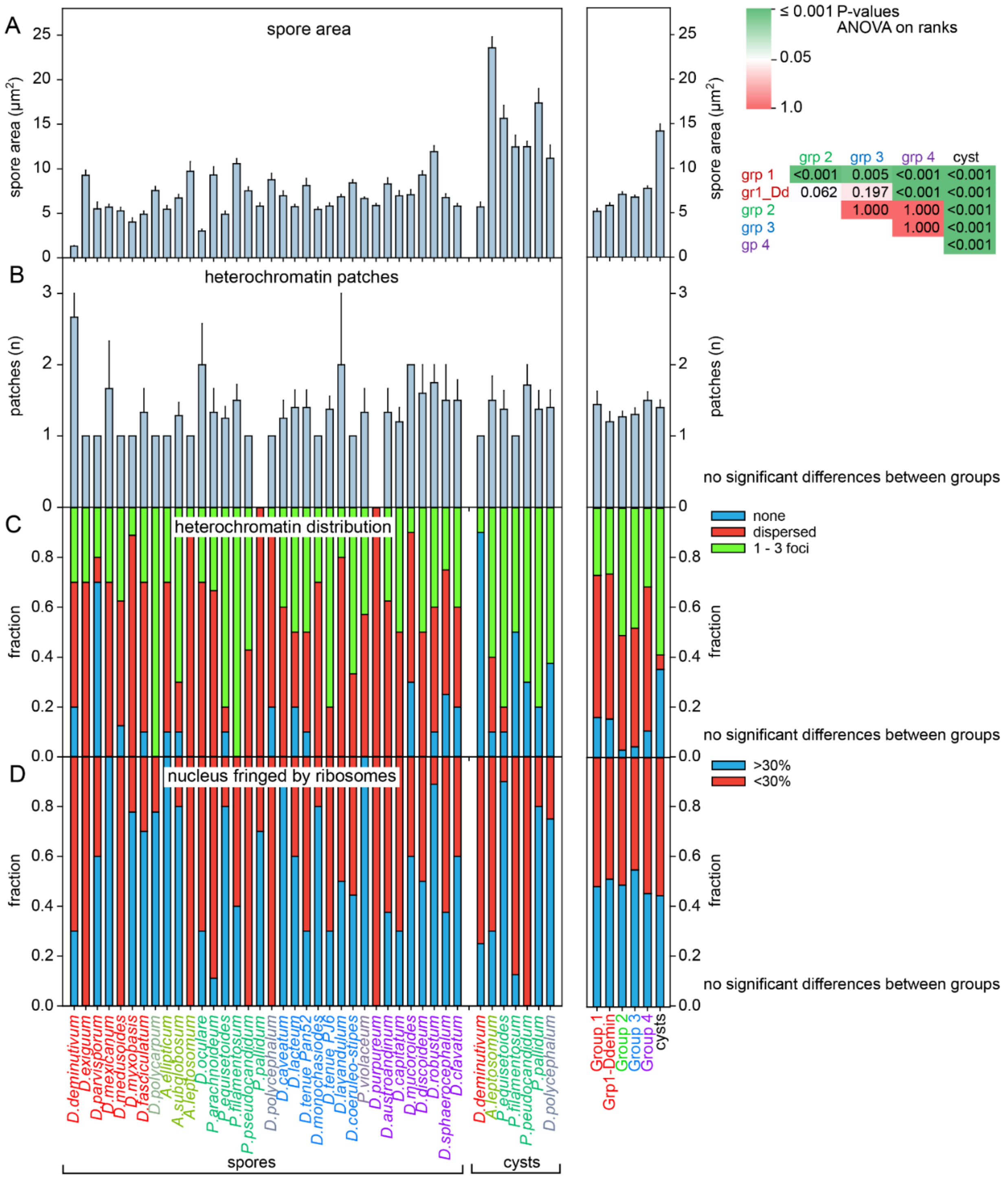

Figure S12. Cell and nuclear features

Cross-section areas of spore and cysts were measured to determine relative contributions of granules and vesicles to cells and show that cysts are significantly larger than spores (A). Both cell types take equally long to differentiate upon starvation, so the spores either go through another round of division or the cysts retain more water than spores. As reported previously group 1 species have relatively small spores [6].

We also measured some nucleus associated features such as the percentage of the circumference of the nucleus that is fringed by ribosomes (D), the distribution pattern of heterochromatin (C) and when present in distinct foci, the number of foci (B). These features show large variation between species and no group-specific trends, except that in cysts heterochromatin was either present in one or two foci, or not visible in the section.

Correlations between spore/cyst fitness and morphology

Survivial conditions	4ºC	-20ºC wet	-20ºC dry	Wall width	Cell area	Granules (#/section)	Granule diameter	Granule (% of cell area)	Vesicles(#/cell)	individual vesicle area	Vesicles total area	Vesicles (% of cell area)	Mitochondria (#/cell)	Crenate mitochondria	Mitochondria with closed cristae	Mitochondria with ribosomes	Granules at poles	Dispersed granules
Pearson Spores and cysts																		
22ºC	0.03	-0.02	-0.07	0.33	0.18	-0.22	0.17	-0.21	0.18	0.23	0.49	0.20	0.17	-0.06	-0.09	-0.18	-0.10	0.03
4ºC		0.65	0.50	0.28	-0.16	-0.06	-0.09	-0.12	-0.06	-0.20	-0.17	-0.15	0.02	0.30	0.15	0.32	0.21	-0.15
-20ºC wet			0.67	0.49	-0.28	-0.16	-0.03	0.03	0.06	-0.38	-0.20	-0.11	-0.12	0.20	0.42	0.53	0.14	-0.04
-20ºC dry				0.39	-0.09	-0.13	-0.03	-0.09	0.14	-0.37	-0.20	-0.25	0.10	0.21	0.29	0.65	0.09	-0.10
Spearman Spores and cysts																		
22ºC	-0.17	-0.09	-0.08	0.34	-0.03	-0.37	0.24	0.01	0.21	0.13	0.34	0.26	0.12	-0.04	-0.09	-0.11	-0.10	0.00
4ºC		0.58	0.60	0.18	-0.13	0.00	-0.05	-0.09	-0.04	-0.13	-0.32	-0.13	-0.01	0.34	0.09	0.40	0.24	-0.11
-20ºC wet			0.77	0.44	-0.20	-0.03	-0.06	-0.04	0.14	-0.22	-0.29	-0.12	-0.08	0.18	0.33	0.48	0.21	0.02
-20ºC dry				0.38	-0.07	-0.10	0.04	-0.06	0.20	-0.33	-0.38	-0.20	0.07	0.21	0.16	0.62	0.28	-0.11
Pearson Spores																		
22ºC	0.15	0.08	0.07	0.30	0.26	-0.17	0.36	-0.05	0.45	-0.03	0.47	0.19	0.31	-0.19	-0.14	-0.14	0.03	0.04
4ºC		0.49	0.35	0.30	0.34	0.34	-0.19	-0.41	-0.06	-0.01	0.01	-0.29	0.29	0.15	0.07	0.23	-0.06	0.08
-20ºC wet			0.56	0.56	0.22	0.32	-0.14	-0.22	0.12	-0.32	-0.02	-0.27	0.15	-0.06	0.42	0.49	-0.22	0.32
-20ºC dry				0.42	0.33	0.19	-0.14	-0.32	0.14	-0.27	-0.05	-0.37	0.35	0.04	0.28	0.62	-0.22	0.14
Spearman Spores																		
22ºC	-0.12	-0.05	0.05	0.33	-0.04	-0.43	0.42	0.08	0.55	-0.11	0.28	0.17	0.23	-0.16	-0.22	-0.06	-0.01	0.01
4ºC		0.34	0.35	0.06	0.09	0.27	-0.17	-0.28	-0.15	-0.01	-0.12	-0.24	0.17	0.23	0.03	0.23	-0.02	0.13
-20ºC wet			0.66	0.38	0.01	0.24	-0.17	-0.22	0.13	-0.16	-0.10	-0.28	0.14	-0.02	0.31	0.38	-0.07	0.32
-20ºC dry				0.41	0.32	0.19	-0.05	-0.28	0.18	-0.24	-0.14	-0.40	0.40	0.01	0.11	0.53	-0.09	0.23

Table S3. Correlation matrix.
Matrix of correlation coefficients (R) between spore/cyst survival under different conditions and ultrastructural features of spores and cysts as reported in figures S8 to S12 and measured across 27 species. The colour coding intensity reflects the value of R, with positive and negative correlations shown in red and blue respectively. Values surrounded by black borders were statistically significant with $P<0.05$. Values were estimated using both Pearson product moment and Spearman rank order correlation using datasets comprising both spores and cysts and spores only. The complete data tables and correlation matrices with P-values are shown in Supplemental Data 4_Correlations.xlsx. The width of spore walls and the presence of mitochondria with closed cristae and/or fringed by ribosomes is positively correlated with spore survival in wet or dry frost, while the relative area occupied by granules and vesicles is negatively correlated.

Distribution of Dictyostelia across climate zones

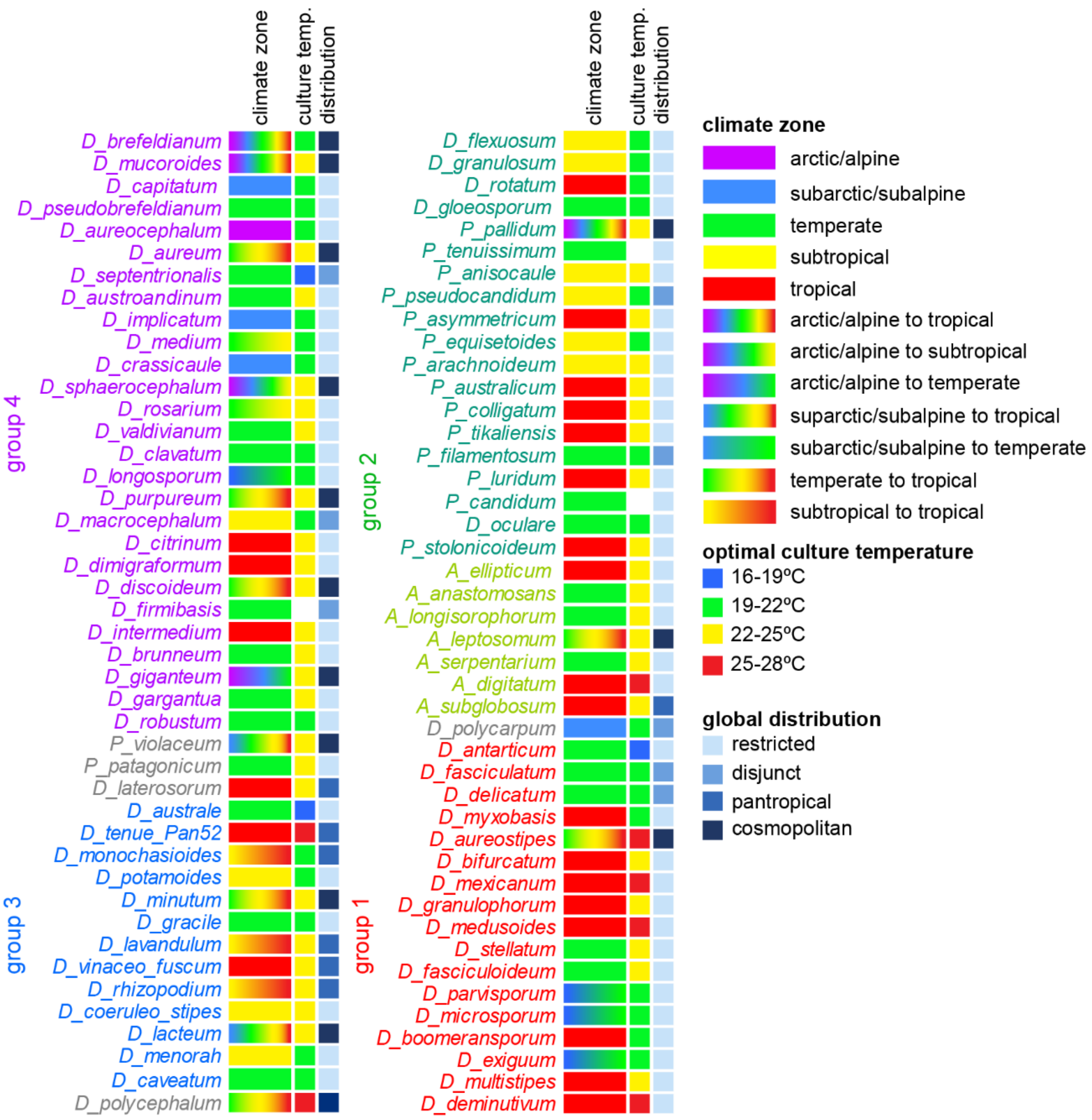

Figure S13. Global distribution of Dictyostelia and optimal culture temperatures
Data on geographical location, distribution and optimal culture temperature were retrieved from original species diagnosis and further studies on species ecology (see supplemental Data5_Ecology.xlxs), and are concisely plotted onto phylogenetically ordered *Dictyostelium* species [6].

Divergence time estimation

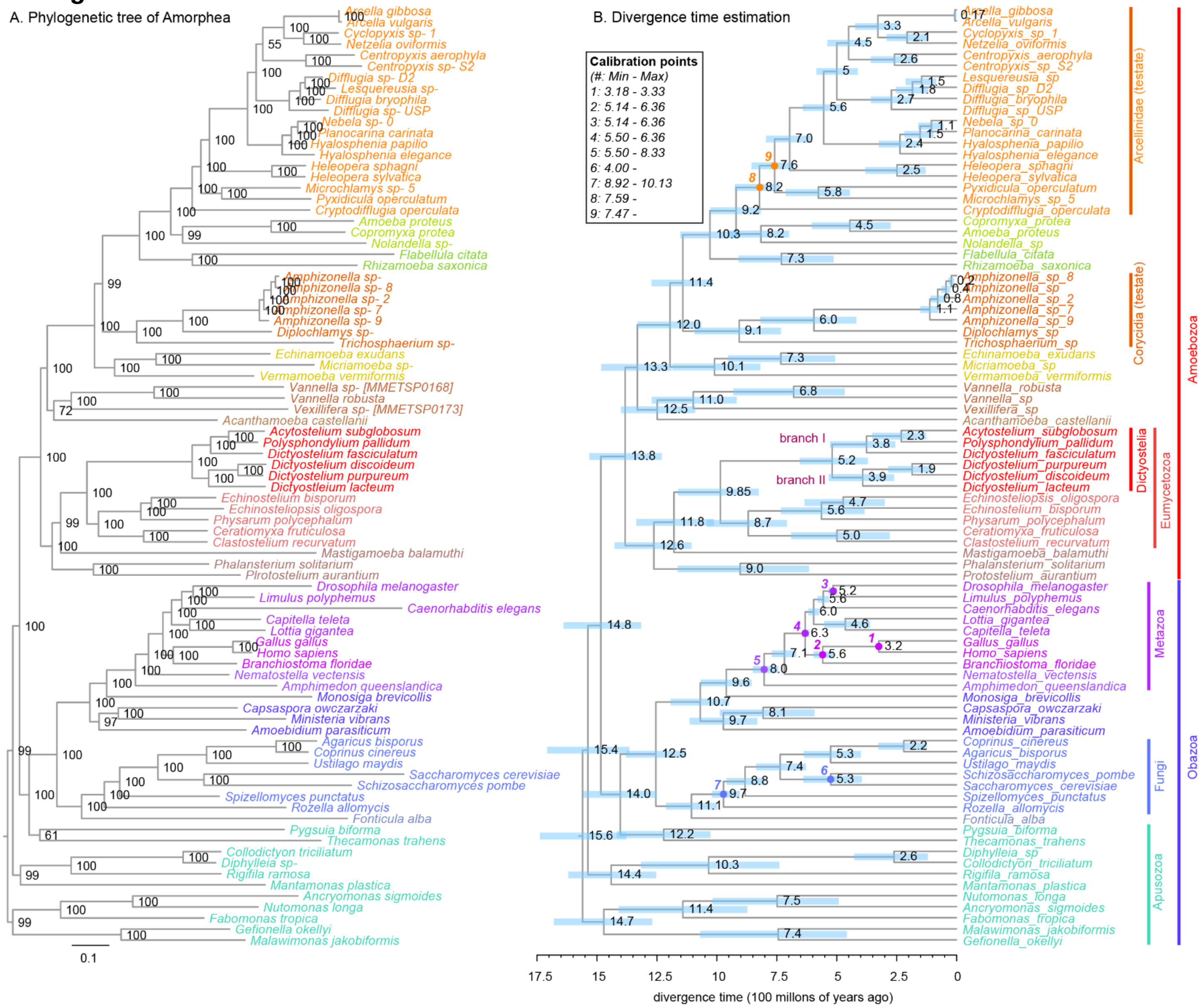

Figure S14. Divergence time estimation

A. Phylogeny of 85 Amorphea. A phylogenetic tree was inferred using IQ tree from concatenated alignment of 240 genes across 85 species, selected to represent the genetic diversity within the group and including Dictyostelid species from each of the four major taxon groups of Dictyostelia. Bootstrap values for the interior nodes are shown.

B. Divergence time estimates. The topology of the phylogeny in panel A and a set of fossil age calibration points in Metazoa, Fungi and testate Amoebozoa (Table S4) were used to infer divergence times at interior nodes of the tree using the MCMCTree software in PAML v. 4.9 [7](See Materials and Methods). The nodes whose estimates were constrained by fossil calibrations are labelled with italic numbers, and the specific minimum and maximum dates that were used are shown in the subset legend (maximums are only shown when available and dates are scaled in 100 mya). The mean estimate of the divergence times in hundred millions of years ago are shown at the nodes, with 95% Highest Posterior Density (HPD) intervals outlined by blue bars. The divergence between the two major branches of Dictyostelia is estimated to have occurred at 520 million years ago.

Table S4: List of fossil calibration points

Taxon	Fossil	Min (Mya)	Max (Mya)	Source
Amniota	*Hylonomus lyelli*	318	333	Fossil Calibration Database[8]
Chordata	*Haikouichtys ercaicunensis*	514	636	Fossil Calibration Database
Arthropoda	*Yicaris dianensis*	514	636	Fossil Calibration Database
Bilateria	*Kimberella quadrata*	550	636	Fossil Calibration Database
Metazoa	*Kimberella quadrata*	550	833	Fossil Calibration Database
Ascomyceta	*Paleopyrenomycites*	400	-	[9]
Fungi	*Ourasphaira giraldae*	892	1013	[10]
Arcellinida	*Cyclocyrillum*	759	-	[11]
Gluttino+Organoconcha (Arcellinida)	*Melanocyrillium*	747	-	[11]

List of dated fossils of species in Metazoa, Fungi, and testate Amoebozoa that were used to calibrate the likely divergence time estimate of the two major branches of Dictyostelia (Fig. S14B and Fig. 5)

Table S5. Divergence time estimates at different prior settings

Clade	Uniform (mya) [95% HPD]	Skew-normal (mya) [95% HPD]	Skew-T (mya) [95% HPD]	Cauchy (mya) [95% HPD]
Dictyostelia	519 [372, 652]	521 [386, 656]	551 [404, 703]	590 [431, 744]
Metazoa	803 [746, 848]	820 [739, 900]	896 [764, 1038]	948 [832, 1086]
Fungi	972 [907, 1018]	963 [892, 1032]	1045 [892, 1267]	1150 [946, 1345]

Divergence times with 95 % HPD intervals of Dictyostelia, Metazoa and Fungi using different prior distributions were generated for nodes with both minimum and maximum constraints as implemented in the R-package “MCMCtreeR” [12]. A uniform distribution was used to generate Figures 5 and S14B).

Supplemental References

1 Sheikh, S. *et al.* A New Classification of the Dictyostelids. *Protist* **169**, 1-28, doi:10.1016/j.protis.2017.11.001 (2018).
2 Schaap, P. *et al.* Molecular phylogeny and evolution of morphology in the social amoebas. *Science* **314**, 661-663 (2006).
3 West, C. M. Comparative analysis of spore coat formation, structure, and function in Dictyostelium. *Int Rev Cytol* **222**, 237-293 (2003).
4 Bomblies, L. *et al.* Membrane-enclosed crystals in Dictyostelium discoideum cells, consisting of developmentally regulated proteins with sequence similarities to known esterases. *J.Cell Biol.* **110**, 669-679 (1990).
5 Hohl, H. R., Miura-Santo, L. Y. & Cotter, D. A. Ultrastuctural changes during formation and germination of microcysts in *Polysphondylium pallidum*, a cellular slime mould. *J. Cell Sci.* **7**, 285-306 (1970).
6 Romeralo, M. *et al.* Analysis of phenotypic evolution in Dictyostelia highlights developmental plasticity as a likely consequence of colonial multicellularity. *Proc Biol Sci* **280**, 20130976, doi:10.1098/rspb.2013.0976 (2013).
7 Yang, Z. H. PAML 4: Phylogenetic analysis by maximum likelihood. *Molecular Biology and Evolution* **24**, 1586-1591, doi:10.1093/molbev/msm088 (2007).
8 Ksepka, D. T. *et al.* The Fossil Calibration Database—A New Resource for Divergence Dating. *Syst. Biol.* **64**, 853-859, doi:10.1093/sysbio/syv025 (2015).
9 Taylor, T. N., Hass, T. & Kerp, H. The oldest fossil ascomycetes. *Nature* **399**, 648-648, doi:10.1038/21349 (1999).

10 Loron, C. C. *et al.* Early fungi from the Proterozoic era in Arctic Canada. *Nature* **570**, 232-+, doi:10.1038/s41586-019-1217-0 (2019).
11 Lahr, D. J. G. *et al.* Phylogenomics and Morphological Reconstruction of Arcellinida Testate Amoebae Highlight Diversity of Microbial Eukaryotes in the Neoproterozoic. *Curr Biol* **29**, 991-1001.e1003, doi:10.1016/j.cub.2019.01.078 (2019).
12 Puttick, M. N. MCMCtreeR: functions to prepare MCMCtree analyses and visualise posterior ages on trees. *Bioinformatics (Oxford, England)*, doi:10.1093/bioinformatics/btz554 (2019).
